# In Vivo Assessment of Thermosensitive Liposomes for the Treatment of Port Wine Stains by Antifibrinolytic Site-Specific Pharmaco-Laser Therapy

**DOI:** 10.3390/pharmaceutics12060591

**Published:** 2020-06-25

**Authors:** Mingjuan Li, M. Ingmar van Raath, Shervin Khakpour, Ahmet Seçilir, Bart C. Sliggers, Xuan Huang, Baoyue Ding, Gert Storm, René R. van der Hulst, Anton I.P.M. de Kroon, Michal Heger

**Affiliations:** 1 Department of Pharmaceutics, Jiaxing Key Laboratory for Photonanomedicine and Experimental Therapeutics, College of Medicine, Jiaxing University, Jiaxing 314001, Zhejiang, China; limingjuan@zjxu.edu.cn (M.L.); ingmarvanraath@gmail.com (M.I.v.R.); hx@zjxu.edu.cn (X.H.); lena_310@zjxu.edu.cn (B.D.); 2 Department of Plastic, Reconstructive, and Hand Surgery, Maastricht University Medical Center, Maastricht University, 3229 HX Maastricht, The Netherlands; r.vander.hulst@mumc.nl; 3 Membrane Biochemistry and Biophysics, Institute of Biomembranes, Utrecht University, 3584 CH Utrecht, The Netherlands; sherwinkh@gmail.com (S.K.); a.secilir@sofabv.nl (A.S.); a.i.p.m.dekroon@uu.nl (A.I.P.M.d.K.); 4 Department of Medical Innovation and Development, Amsterdam University Medical Centers, location AMC, University of Amsterdam, 1105 AZ Amsterdam, The Netherlands; b.c.sliggers@amsterdamumc.nl; 5 Department of Pharmaceutics, Utrecht Institute for Pharmaceutical Sciences, Utrecht University, 3584 CG Utrecht, The Netherlands; g.storm@uu.nl

**Keywords:** tranexamic acid, endovascular laser-tissue interactions, targeted drug delivery, vascular malformations, hyperthermia, thrombosis, fluorescent thrombus staining, intravital fluorescence microscopy

## Abstract

Antifibrinolytic site-specific pharmaco-laser therapy (SSPLT) is an experimental treatment modality for refractory port wine stains (PWS). Conceptually, antifibrinolytic drugs encapsulated in thermosensitive liposomes are delivered to thrombi that form in semi-photocoagulated PWS blood vessels after conventional laser treatment. Local release of antifibrinolytics is induced by mild hyperthermia, resulting in hyperthrombosis and complete occlusion of the target blood vessel (clinical endpoint). In this study, 20 thermosensitive liposomal formulations containing tranexamic acid (TA) were assayed for physicochemical properties, TA:lipid ratio, encapsulation efficiency, and endovesicular TA concentration. Two candidate formulations (DPPC:DSPE-PEG, DPPC:MPPC:DSPE-PEG) were selected based on optimal properties and analyzed for heat-induced TA release at body temperature (T), phase transition temperature (T_m_), and at T > T_m_. The effect of plasma on liposomal stability at 37 °C was determined, and the association of liposomes with platelets was examined by flow cytometry. The accumulation of PEGylated phosphocholine liposomes in laser-induced thrombi was investigated in a hamster dorsal skinfold model and intravital fluorescence microscopy. Both formulations did not release TA at 37 °C. Near-complete TA release was achieved at T_m_ within 2.0–2.5 min of heating, which was accelerated at T > T_m_. Plasma exerted a stabilizing effect on both formulations. Liposomes showed mild association with platelets. Despite positive in vitro results, fluorescently labeled liposomes did not sufficiently accumulate in laser-induced thrombi in hamsters to warrant their use in antifibrinolytic SSPLT, which can be solved by coupling thrombus-targeting ligands to the liposomes.

## 1. Introduction

Port wine stains (PWS) are congenital lesions in the skin that are comprised of ectatic venule-like vasculature [1,2]. Due to extensive density and abnormally large blood volume, the hyperdilated blood vessels cause the skin to appear red-to-purple [3]. Since the mid-1980s, the standard treatment of PWS has been pulsed dye laser therapy, which non-invasively induces photothermal damage to PWS vasculature by a process called selective photothermolysis [4]. Hemoglobin is exploited as a blood vessel-confined target chromophore to generate intralumenal heat and thermally coagulate blood without notable extravascular damage [5,6]. Completely photocoagulated blood vessels are subsequently removed by wound healing mechanisms and replaced by normal-sized capillaries, which coincides with a reduction in vascular density, blood volume, and lesional redness [7]. However, these processes are only effective in completely photocoagulated blood vessels [8], achieved in approximately half of the laser-treated patients [9,10]. The incompletely photocoagulated PWS vasculature is responsible for therapeutic recalcitrance and suboptimal clinical outcomes [7,11]. The PWS patients with poorly responding and refractory lesions do not have alternative treatment options, while a substantial fraction of these patients desire more effective therapies to become available [12]. On top of that, the numerous innovations and ancillary interventions in laser treatment of PWS have not translated to improved clinical outcomes during the last three decades [9,10], altogether creating a strong medical need for novel, more effective treatment modalities [10].

**Figure 1:**
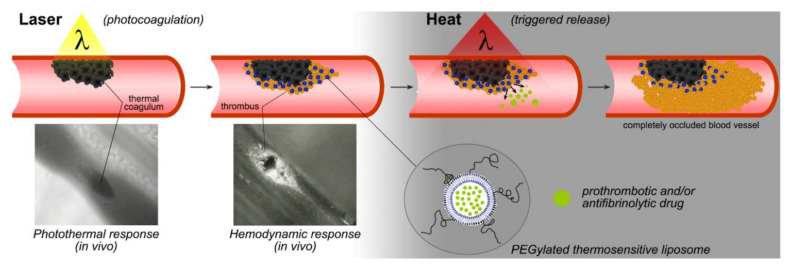
Theoretical principles of site-specific pharmaco-laser therapy (SSPLT) in the context of incompletely photocoagulated port wine stain (PWS) vessels, which are responsible for therapeutic recalcitrance. The processes are divided into conventional laser treatment by selective photothermolysis (white background) and the additional procedures of SSPLT (gray background). PWS vasculature is illuminated with a yellow laser pulse (λ), leading to the photocoagulation of blood and the formation of a thermal coagulum. Thermal coagula are inherently pro-thrombotic and therefore trigger coagulation and platelet aggregation at the thermal coagulum interface. In time, the thrombi dislodge or break down and the PWS vessels remain only partially occluded. In SSPLT, thrombus-targeting thermosensitive liposomes containing anti-fibrinolytic and/or prothrombotic agents are systemically administered directly before conventional laser treatment to ensure that the highest possible amount of liposomes is present in the circulation during lasing. Upon encountering a laser-induced thrombus, the liposomes accumulate in the developing clot. Next, a second laser emitting red light (λ) is used to induce mild hyperthermia in PWS skin, causing the phospholipid bilayer to undergo phase transition and release the encapsulated drugs that locally exacerbate the degree of thrombosis to the extent of entirely occluding the vascular lumen (clinical endpoint).

To satisfy that need, we devised site-specific pharmaco-laser therapy (SSPLT, Figure 1) [7,9,11,13,14]. SSPLT is a development-stage treatment modality that aims to utilize targeted photonanomedicines to modulate the biological responses to endovascular laser–tissue interactions in order to accomplish complete occlusion of PWS vasculature [15], which marks the clinical endpoint. Laser-irradiated blood vessels contain a thermal coagulum [5,6] that is prothrombotic in nature in partially occluded blood vessels [15]. The laser-mediated thrombosis can be exacerbated pharmacologically to culminate in complete vaso-occlusion [7,9,11] and meet the biological prerequisites for complete clearance. The degree of thrombosis can be augmented by deterring thrombus breakdown (fibrinolysis) through the use of antifibrinolytics, which is dubbed antifibrinolytic SSPLT. Alternatively, the size of the thrombus can be expanded with prothrombotics, referred to as prothrombotic SSPLT. Both antifibrinolytics and prothrombotics have to be released/activated at the site of laser-induced endovascular damage so as not to systemically disrupt hemostatic homeostasis.

For antifibrinolytic SSPLT, we formulated tranexamic acid (TA)-containing thermosensitive PEGylated DPPC and DPPC:lysoPC liposomes [16] that are envisaged to target to laser-induced thrombi and release TA upon a mild hyperthermic stimulus [17] using near-infrared light [9,11,14]. TA is a potent antifibrinolytic drug used clinically to deter heavy bleeding during e.g., surgery [18,19] through its inhibition of the enzyme (plasmin) responsible for thrombolysis [20]. To date our group has demonstrated that non-encapsulated TA reduces laser-induced thrombus breakdown in hamsters (manuscript in preparation). When TA is loaded into abovementioned formulations, the liposomes release nearly all encapsulated TA within 2.5 min when heated to phase transition temperature (T_m_) in buffered solution. Based on the physicochemical properties of the liposomes, in vitro TA release kinetics, and anatomical features of PWS vascular architecture [16,21,22], it was calculated that roughly 10^5^–10^8^ liposomes are required to instill a pharmacologically relevant antifibrinolytic effect in a 500-µm long vessel segment with a diameter of 30–200 µm [8]. We further established that PEGylated DPPC liposomes exhibit weak association with resting and activated platelets [16,23,24], which spawned the hypothesis that such association between platelets and liposomes may be sufficient to deliver ample liposomal TA to laser-induced thrombi for an antifibrinolytic effect.

The main aim of this study was therefore to assess the feasibility of using PEGylated thermosensitive liposomes for antifibrinolytic SSPLT without thrombus-specific (immuno)targeting. First, 14 novel thermosensitive formulations were evaluated for TA:lipid ratio, encapsulation efficiency (E_eff_), and endovesicular TA concentration (*C*_TA_) and benchmarked against the previously established candidate formulations DPPC:DSPE-PEG (96:4 molar ratio) and DPPC:MPPC:DSPE-PEG (86:10:4). Since none of the newly tested formulations proved superior to the benchmark formulations, DPPC:DSPE-PEG (96:4) and DPPC:MPPC:DSPE-PEG (86:10:4) were investigated further for content release kinetics at body temperature, at T_m_, and at temperatures (T) > T_m_ in buffer and in the presence of increasing fractions of human plasma. Finally, the incorporation of PEGylated thermosensitive liposomes into laser-induced thrombi was studied using a validated hamster dorsal skinfold model in combination with intravital fluorescence microscopy.

## 2. Materials and Methods

All concentrations listed throughout this manuscript refer to final concentrations (fc) unless indicated otherwise. Online Supplementary Material is designated with a prefix ‘S’. A list of abbreviations is provided in Supplementary Section S1.

### 2.1. Materials

All chemicals and reagents used are listed in Table S1. Phospholipids were dissolved in CHCl_3_ and stored at −20 °C under a nitrogen atmosphere in the dark. Phospholipid stock concentrations were determined by an inorganic phosphorous assay [16] modified from Rouser et al. [25]. Fluorescamine was dissolved in acetone at a 1.08-mM stock concentration [26] and stored at 4 °C under a nitrogen atmosphere in the dark. Liposomes were stored at 4 °C under a nitrogen atmosphere in the dark for a maximum of 14 d. Based on osmolarity measurements (Supplementary Section S2.1), a solution containing 318 mM TA and 10 mM HEPES in MilliQ, pH = 7.4 (adjusted with 37% HCl), and an osmolarity of 0.304 osmol·kg^−1^ was used for TA-encapsulating liposome preparation. The iso-osmolar physiological buffer, which was used as diluent and eluent in all experiments, consisted of 10 mM HEPES and 150.6 mM (0.88% *w*/*v*) NaCl, pH = 7.4, 0.291 osmol·kg^−1^.

### 2.2. Tranexamic Acid-Encapsulating Liposome Preparation and Characterization

TA-encapsulating large unilamellar vesicles prepared by the extrusion technique (LUVETs) were formulated at the following compositions: formulation *F1*, DPPC (100); *F2*, DPPC:DSPE-PEG2000 (98:2); *F3*, DPPC:DSPE-PEG2000 (96:4); *F4*, DPPC:DSPE-PEG2000 (94:6); *F5*, DPPC:MPPC (90:10); *F6*, DPPC:MPPC:DSPE-PEG2000 (86:10:4); *F7*, DPPC:DOPE-PEG2000 (98:2); *F8*, DPPC-DOPE-PEG2000 (96:4); *F9*, DPPC:DOPE-PEG2000 (94:6); *F10*, DPPC:DOPE-PEG5000 (98:2); *F11*, DPPC:DOPE-PEG5000 (96:4); *F12*, DPPC:DOPE-PEG5000 (94:6); *F13*, DPPC:SDPC:DSPE-PEG2000 (95:1:4); *F14*, DPPC:SDPC:DSPE-PEG2000 (91:5:4); *F15*, DPPC:SDPC:DSPE-PEG2000 (86:10:4); *F16*, DPPC:SDPC:DSPE-PEG2000 (93:1:6); *F17*, DPPC:SDPC:DSPE-PEG2000 (89:5:6); *F18*, DPPC:SDPC:DSPE-PEG2000 (84:10:6); *F19*, DPPC:MPPC:SDPC:DSPE-PEG2000 (81:10:5:4); and *F20*, DPPC:MPPC:SDPC:DSPE-PEG2000 (79:10:5:6). *F3* and *F6* are candidate formulations from our previous work [16] that serve as benchmarks in this study and are conveniently referred to as DPPC:DSPE-PEG and DPPC:MPPC:DSPE-PEG, respectively, throughout the text.

TA-encapsulating LUVETs were prepared as described in [16]. Briefly, phospholipids in CHCl_3_ were mixed at the desired ratios. The solution was desiccated by evaporation under a stream of N_2_ gas and desiccated for 20 min in a vacuum desiccator at room temperature (RT). The resulting lipid film was hydrated with 318 mM TA in 10 mM HEPES buffer (pH = 7.4, 0.301 osmol·kg^−1^ based on the linear fit function, Supplementary Section S2.1) to a lipid concentration of 5 mM and bath sonicated for 10 min. The mixture was subjected to 10 freeze-thaw cycles and extruded 5× through 0.2-μm Anopore aluminum oxide filters (Anotop, Whatman, Brentford, UK) at 55 °C (thermoregulated water bath).

Unencapsulated TA was removed from the LUVET suspensions by size exclusion chromatography during 4-min centrifugation at 100× *g* and 4 °C. Chromatography columns consisted of a 2-mL plastic syringe (Becton Dickinson, Franklin Lakes, NJ, USA) containing a gel volume of 2.2–2.5 mL (Sephadex G-50 fine, GE Healthcare, Chalfont St. Giles, UK). The Sephadex was suspended in physiological buffer and stored at 4 °C under nitrogen gas for at least 24 h before use. Before sample loading, the column was dried by centrifugation at 900× *g* for 4 min at 4 °C. The loading volume was 200 μL. All chromatography and storage steps were performed on ice to deter incidental TA leakage from the LUVETs.

Phospholipid concentration in eluted LUVET suspensions was determined by a phosphorous assay following perchloric acid digestion of phospholipids [16], modified from Rouser et al. [25]. Encapsulated TA was quantitated spectrofluorometrically as described in Section 2.3. LUVET size, polydispersity index, and zeta potential (selected samples) were determined by photon correlation spectroscopy and electrophoretic mobility measurements (Zetasizer 3000, Malvern Instruments, Malvern, UK) at instrument settings as specified in Supplementary Section S2.2 and in [27]. Particle size was considered monodisperse at a polydispersity index of ≤ 0.3 [28]. For all measurements, LUVETs were diluted with physiological buffer (RT). Five consecutive runs of 10 iterations were performed to determine size and polydispersity index and the values were averaged by the software.

Phospholipid phase transition temperatures (T_m_) were measured by differential scanning calorimetry (MicroCal, Northampton, MA, USA) after the dilution of LUVETs with ice-cold physiological buffer to a 3-mM final lipid concentration. Instrument settings are provided in Supplementary Section S2.2. The physiological buffer was used as a reference. Values (delta heat flow) were background-corrected and normalized to maximum. For internal validation and reference purposes, liposomes were prepared from pure DPPC in water as well as pure DSPC in water as described above but without the bath sonication step.

### 2.3. Calculation Drug: Lipid Ratio, Encapsulation Efficiency, Trapped Volume, and Endovesicular Tranexamic Acid Concentration

The calculation method for TA:lipid ratio, E_eff_, trapped volume (V_t_), and *C*_TA_ have been published previously [16] but are reiterated and, where necessary, modified here for easy reader access and data interpretation.

Drug:lipid ratios were calculated by dividing the TA concentration as determined by the fluorescamine assay by the phospholipid concentration as determined by the phosphorous assay (Section 2.2). The TA encapsulated in LUVETs was quantified spectrofluorometrically following derivatization with fluorescamine as described previously [16]. After gelfiltration (Section 2.2), the LUVETs were gently homogenized with a pipette in the eluate and diluted 500× with ice-cold physiological buffer. Next, 500 μL of the diluted LUVET solution was mixed with 250 μL of 5% TX100 (1% fc) and 500 μL of 1.08 mM fluorescamine in acetone (432 μM fc) in a 1.5-mL Eppendorf tube (final dilution factor of 1250). Following 30 min of incubation at 37 °C in an orbital shaker (Thermomixer, Eppendorf|Fisher Scientific, Hamburg, Germany), the samples were assayed spectrofluorometrically at λ_ex_ = 391 ± 5 nm and λ_em_ = 483 ± 5 nm (SPF 500C, American Instrument Company, Silver Springs, MD, USA). Standards in the 0–4 μM TA concentration range (diluted from a 318-mM stock solution in physiological buffer) were included in each separate experiment for calibration. The liposomal TA concentration was calculated using the regression equation of the calibration curve. This protocol was used to determine TA:lipid ratios and to quantify heat-induced TA release in buffer.

The E_eff_ was computed by dividing the liposomal TA:lipid molar ratio by the initial TA:lipid molar ratio (318 mM TA per 5 mM phospholipid, i.e., 63.6) and expressed as a percentage.

The V_t_ (L·mole^−1^ lipid) was computed with the equation obtained from [29]:

(1) $$V_{t}=\left(500\;/\;3\right)\times \left(A\right)\times \left(N\right)\times (r_{v})$$

where A is the area of the membrane occupied by one lipid, N is the Avogadro constant (6.022 × 10^23^ mol^−1^), and r_v_ the radius of the vesicle (based on photon correlation spectroscopy, Section 2.2). The area per phospholipid molecule used in the calculations is 49.4 Å^2^ for DPPC [30], 50.0 Å^2^ for DSPE-PEG [31], 48.0 Å^2^ for MPPC [32], 58.0 Å^2^ for SDPC [33], and 67.0 Å^2^ for DOPE-PEG [34]. For phospholipid mixtures, the areas were weighed averages indexed for the molar ratio of each lipid component:

(2) $$A_{weighed}=\frac{\left[\begin{array}{c} \left(A_{DPPC}\right)\left({\mathrm{mol}\%}_{DPPC}\right)+\left(A_{DSPE-PEG2000}\right)\left(mol{\%}_{DSPE-PEG2000}\right)+\left(A_{MPPC}\right)\left(mol{\%}_{MPPC}\right)+\\ \left(A_{DOPE-PEG2000}\right)\left(mol{\%}_{DOPE-PEG2000}\right)+\left(A_{DOPE-PEG5000}\right)\left(mol{\%}_{DOPE-PEG5000}\right) \end{array}\right]}{100\%}$$

The V_t_ per vesicle (eV_t_, expressed in L/vesicle) was derived by extrapolating the quantity of phospholipid molecules per vesicle. The quantity of phospholipid molecules per vesicle was defined as the cumulative number of lipids in the outer (l_om_) and inner membrane leaflet (l_im_), based on the *A_weighed_*, the measured vesicle size with radius r_v_, a bilayer thickness of 3.93 nm [32], and a spherical morphology (where area sphere = 4πr^2^):

(3) $$I_{om}=4\pi v^{2}/A_{weighed}$$

(4) $$I_{im}=4\pi \times {\left(r_{v}-3.93\right)}^{2}/A_{weighed}$$

The *eV_t_* was calculated by:

(5) $$eV_{t}=\left[\left(I_{om}+I_{im}\right)\times V_{t}\right]/N$$

The quantity of *TA* molecules per vesicle (*Q_TA_*) was obtained by multiplying (*l_om_* + l_im_) by the TA:lipid ratio. Subsequently, the *C*_TA_ was computed from the amount of TA molecules per vesicle for a given *eV_t_*:

(6) $$C_{TA}=\left(Q_{TA}/N\right)\times \left(1/eV_{t}\right)$$

### 2.4. Heat-Induced Tranexamic Acid Release from Thermosensitive LUVETs in Physiological Buffer

Heat-induced drug release from TA-encapsulating DPPC:DSPE-PEG (96:4) (*F3*, Section 2.2) and DPPC:MPPC:DSPE-PEG (86:10:4) (*F6*, Section 2.2) LUVETs was investigated as described before [16,17]. The non-diluted eluent from the first chromatography step (Section 2.2) was subjected to a second size exclusion chromatography step following an identical protocol to ensure complete removal of unencapsulated TA.

Prior to heat treatment, the gelfiltered LUVET suspensions were diluted 10× with ice-cold physiological buffer. From this diluted LUVET suspension, aliquots of 20 μL were diluted 50-fold (N = 3 per experiment) in ice-cold physiological buffer and assayed spectrofluorometrically for total liposomal TA concentration as described in Section 2.3 (final dilution factor of 1250). The mean total liposomal TA concentration was used to calculate the percentage of released TA molecules after a hyperthermic stimulus.

Following 5-min equilibration at 4 °C, 160 μL of the 10-fold diluted LUVETs was transferred to 0.2 mL ultra-thin PCR tubes (Thermowell Gold, Corning, New York, NY, USA) and incubated at 4 °C for 10 min before thermally-induced TA release, which was carried out in a thermal cycler (Biozym, Oldendorf, Germany). Samples to determine 100% TA content were transferred to ultra-thin PCR tubes but kept at 4 °C. LUVETs were heated at a pre-set temperature for a predefined period, after which they were immediately cooled in an ice bath for at least 2 min. DPPC:DSPE-PEG (96:4) LUVETs were exposed to 37.0, 39.3, 42.3, 43.3, 47.3, 65.0, and 90.0 °C, whereas DPPC:MPPC:DSPE-PEG (86:10:4) LUVETs were exposed to 36.0, 37.0, 40.0, 41.5, 44.0, 46.5, 65.0, and 90.0 °C. The entire volume was then transferred to 0.5-mL polycarbonate ultracentrifuge tubes and centrifuged (Optima TLX Ultracentrifuge, Beckman-Coulter, Fullerton, CA, USA) at 355,000× *g* for 60 min at 4 °C to pellet the LUVETs. Four untreated 160-μL LUVET samples were included in the ultracentrifugation step to serve as control for centrifugation-induced TA release. Finally, 50 μL of the supernatant in the ultracentrifuge tubes was carefully aspirated from the top of the solution and transferred to a 15-mL polystyrene centrifuge tube (Corning) containing 2450 μL of physiological buffer (50-fold dilution). Similarly, 50 μL of the control LUVETs (not heated) was transferred into 2450 μL of physiological buffer. TA content was quantitated as described in Section 2.3, again taking into account a final dilution factor of 1250. The experimental workflow is illustrated in Figure S2.

TA release was calculated by dividing the mean TA concentration in the supernatant of heat-treated samples by the mean total TA concentration in the control LUVETs. TA concentrations were corrected for the mean TA content in the supernatant of the ultracentrifugation control samples and plotted as percentage release (relative to mean total TA concentration) as a function of heating time. The mean ± SD TA concentration in the supernatant of the centrifugation control samples of DPPC:DSPE-PEG (96:4) and DPPC:MPPC:DSPE-PEG (86:10:4) LUVETs was 2.4 ± 5.1% (N = 48) and 7.1 ± 11.1% (N = 56) of the total liposomal TA content, respectively, as reported previously [16]. The release rate was calculated as the percentage of TA released at time point X divided by X (i.e., from the onset of heating) and expressed as %·s^−1^. Time point X marked the time at which a plateau in released TA had been reached. Unlike in our previous study [16], the heating lag during the first 0.5 min of heating was accounted for in the calculations, as this is representative of the clinical scenario.

To ensure reproducibility of the data, the experiments were performed by 3 researchers in an independent manner. The presented data represent pooled values of all experiments.

### 2.5. Calcein-Encapsulating Liposome Preparation and Characterization

A self-quenched calcein solution was prepared for real-time spectrofluorometric analysis of heat-induced release from LUVETs. Calcein (1556 mg) was added to 45 mL of MilliQ water and the suspension was heated to 80 °C in a water bath. Next, 1.3 mL of 10 M NaOH in MilliQ water was added to completely dissolve the calcein, after which the pH was gradually lowered to 7.4 by stepwise addition of 1 M HCl (1040 µL total) on a heating plate with magnetic stirring and online pH measurement. The final calcein concentration was 49.6 mM, which had an osmolarity of 0.210 osmol·kg^−1^ (Supplementary Section S2.1). This solution was used to prepare LUVETs containing calcein in a hypo-osmolar aqueous compartment. To render the calcein solution iso-osmolar, 0.26% (*w*/*v*) of NaCl was added to an aliquot of the hypo-osmolar calcein based on the osmolarity measurements (Supplementary Section S2.1).

DPPC:DSPE-PEG (96:4) and DPPC:MPPC:DSPE-PEG (86:10:4) LUVETs containing calcein were prepared at a 5 mM final phospholipid concentration and characterized as described in Section 2.2. The phospholipid films were hydrated with either 49.6 mM calcein solution, pH = 7.4 or with 49.6 mM calcein, 0.23% NaCl, pH = 7.4 to prepare LUVETs with a hypo-osmolar or iso-osmolar aqueous compartment, respectively, relative to normophysiological conditions. Size-exclusion chromatography was performed twice, as described in Section 2.2, to completely remove unencapsulated calcein using 10 mM HEPES and 0.62% NaCl buffer, pH = 7.4, 0.210 osmol·kg^−1^ or physiological buffer as elution buffers for experiments with hypo-osmolar LUVETs and iso-osmolar LUVETs, respectively. In previous experiments, ± 30% retention of LUVETs in the gel matrix was measured after the second gelfiltration step (unpublished results), which was taken into account when reporting final phospholipid concentrations in the calcein release assays.

### 2.6. Stability of Thermosensitive Liposomes in Human Plasma

Human platelet-poor plasma (PPP) was prepared as described in [23] and stored at 4 °C in the dark for a maximum of 7 d. Real-time measurement of calcein release from thermosensitive liposomes was adapted from [23] and performed in a spectrofluorometer equipped with Peltier temperature control of the cuvette holder and magnetic stirring function (Cary Eclipse, Varian, Palo Alto, CA, USA). The cuvette holder, containing a cuvette for magnetic stirrers (quartz glass Suprasil, 10 × 4 mm light path, Hellma Analytics, Müllheim, Germany) was set to 37 °C or 4 °C. PPP and physiological buffer were incubated in a 37 °C stove or at 4 °C for at least 30 min prior to sample preparation. After addition of physiological buffer and PPP to the cuvette at predefined ratios (0%, 20%, 40%, 60%, 80%, and 100% PPP in a final volume of 1485 µL) and homogenization with a pipette, spectrofluorometry was started in kinetics mode (t = 0 min). Calcein fluorescence was measured at λ_ex_ = 488 ± 5 nm and λ_em_ = 522 ± 5 nm. At t = 2 min, 15 µL of the LUVETs was added (35 µM final phospholipid concentration), followed by the addition of 15 µL of 10% TX100 (0.1% fc) at t = 5 min. At t = 7 min, acquisition was stopped.

The release rate (*R*, in %·min^−1^) was calculated from the fluorescence traces by Equation (7), adopted from [23]:

(7) $$R=\left[\frac{\left(F_{t=4.904}-F_{t=3.004}\right)}{\left(F_{ave\left(t=6.663-7.004\right)}-F_{t=1.954}\right)}\times 100\%\right]$$

where *F* is the fluorescence intensity (in arbitrary units, a.u.), t is the time point (in min), and *F_ave_* is the mean fluorescence intensity over the indicated time interval (in min). Data were normalized to the fluorescence emission intensity at 7 min (end of fluorescence acquisition; 2 min after addition of TX100).

To investigate the effect of osmolarity on membrane stability and release, DPPC:DSPE-PEG (96:4) and DPPC:MPPC:DSPE-PEG (86:10:4) LUVETs containing a hypo-osmolar calcein cargo (49.6 mM, pH = 7.4, 0.210 osmol·kg^−1^, Supplementary Section S2.1) were prepared as described in Section 2.5 at 5 mM final phospholipid concentration and subjected to rapid heating while measuring real-time fluorescence (i.e., release) in a repurposed Roche LightCycler 480 II system setup (Roche Molecular Systems, Pleasanton, CA, USA). Size-exclusion chromatography was performed twice on LUVETs as described in Section 2.2 using 10 mM HEPES and 0.62% NaCl buffer, pH = 7.4, 0.210 osmol·kg^−1^ as elution buffer (loading volume: 150 µL). The eluted LUVETs were pooled and kept on ice, along with the freshly prepared PPP that had been diluted with ice-cold physiological buffer to 20% and 60% concentration. Next, the LUVETs were added to the buffer/PPP solution (2:48 volume ratio for DPPC:DSPE-PEG LUVETs, 0.14 mM final phospholipid concentration, and 5:45 volume ratio for DPPC:MPPC:DSPE-PEG LUVETs, 0.35 mM final phospholipid concentration) and aliquoted into wells of a 96-wells PCR plate (50 µL/well, N = 8 per group, Roche Molecular Systems) that had been kept on ice. The plate was sealed with a plastic foil and centrifuged (859× *g*, 5 min, 4 °C) to remove air bubbles. The samples were then thermally insulated with 140 µL of ice-cold vegetable oil (by very gentle pipetting), which proved necessary from preceding pilot experiments to minimize premature calcein release. Thirty min prior to the experiment, the heating block and the top plate of the LightCycler 480 II device were removed and stored in a cold room (4 °C) and reinserted directly before the start of the run to further minimize calcein leakage (Figure S5). The lid is standardly pre-heated by the device before the start of a run; vegetable oil insulation and hardware cooling were therefore implemented to enable the experiment and to improve results. The SybrGreen program in the LightCycler 480 software (λ_ex_/λ_em_ = 460/510 nm), normally used for qRT-PCR [35], was reconfigured manually to maintain 20 °C from 0 to 18 s and then ramp up the temperature at maximum rate to 47.0 °C for up to 80 s. After the experiment, the data were exported in CVS format and plotted in Microcal Origin (OriginLab, Northampton, MA, USA) to render temperature-% release graphs. The percentage release was calculated based on the fluorescence intensity at the end of the experiment (maximum release) following subtraction of background fluorescence at the start of the run.

### 2.7. Animals and Surgical Procedures

Animal experiments were approved by the institutional review board for animal research at the Academic Medical Center, University of Amsterdam (protocol BEX 102550, approval granted on 21 November 2011), which was transferred to Jiaxing University to complete the in vivo study. Animals were treated in compliance with institutional guidelines and the National Institute of Health Guidelines for the Care and Use of Laboratory Animals (8th edition). The animals were used in conformity with the 3 Rs principle [36] in regard to reduction, in that clearly defined stop criteria were implemented in case of negative results and a minimum number of animals was used to establish positive proof-of-concept and obtain results. Male Gold Syrian hamsters (N = 13, 93–122 g) were purchased from Harlan (Belton, UK) and Changzhou Cavens Laboratory Animals (Changzhou, Jiangsu, China) and housed under standardized laboratory conditions with 12-h light/dark cycles and ad libitum access to standard chow (Harlan, Zeist, the Netherlands; Jiangsu Xietong Pharmaceutical Bio-Engineering Company, Nanjing, Jiangsu, China) and water. The animals were acclimated for at least 7 days before entering into an experiment.

Animals that were included in intravital microscopy experiments received buprenorphine (Temgesic, 0.03 mg/kg, 150 µL of 0.03 mg/mL, Schering-Plough, Kenilworth, NJ, USA) subcutaneously as a preoperative analgesic at least 3 h before surgery. In all experiments, a mixture of O_2_/air with 1.5–3.0% isoflurane (Forene, Abbott Laboratories, Queensborough, UK) was used to induce (1:1 L/min O_2_:air) and maintain (0.3:0.7 L/min O_2_:air) general anesthesia, based on the animal’s sedation depth and response to pain stimuli. Induction anesthesia was administered in a red Perspex animal cage connected to the anesthesia tower. Pain stimuli (pinching of paws with tweezers) were administered before every invasive step and the level of isoflurane was adjusted when necessary to ensure proper sedation depth.

Anesthetized hamsters were placed on a custom-built heating stage to maintain body temperature, which was measured with a rectal temperature probe connected to the anesthesia tower (Philips Healthcare, Amsterdam, The Netherlands). The heating stage was comprised of 2 550 × 400 × 2 mm aluminum plates assembled with an 8-mm distance between the plates. In the interplate space, silicone tubing (8 mm and 5 mm outer and inner diameter, respectively) was densely interlaced. Both ends of the silicone tubing were connected to a temperature-controlled circulator (model TLC 2, Tamson Instruments, Bleiswijk, The Netherlands) in a closed loop configuration that pumped a mixture of water and anti-freeze across the heating stage. The temperature on the surface of the stage was measured with a thermistor-based thermometer (Hanna Instruments, Woonsocket, RI, USA) and maintained at 36–38 °C. A red light heating lamp was additionally used to maintain the temperature of animals in the desired range. Animal surgery was performed on the heating stage.

For blood collection for flow cytometry experiments, the jugular vein (N = 3 hamsters) was mobilized (Figure S3A) and severed with surgical scissors. The blood was allowed to freely flow and was collected as described further in Section 2.8, ultimately leading to animal death by exsanguination.

To infuse solutions for intravital microscopy (Section 2.9), the jugular and subclavian veins were exposed by excision of the overlaying portion of thoracic skin [15] (Figure S3). Solutions were injected via the subclavian vein at the subclavian/jugular vein junction that was approached from the animal’s flank at a sharp angle through fascia and fat to prevent bleeding after needle retraction. A 1-mL insulin syringe (BD Biosciences, Franklin Lakes, NJ, USA) was used to inject solutions because it holds no dead volume. The needle was bent at a ~60-degree angle 3 mm from the base to facilitate injection. After injection, an optical chamber was implanted as described previously [15]. Briefly, the dorsal fur was shaved with veterinary clippers and the shaved skin was sutured between 2 steel frames containing an annular window (Figure S3). Part of the skin on one side was excised to expose the dorsal vasculature. Perivascular fat around the target venule was carefully trimmed with 8-mm microsurgical scissors (Liber Medical, Vreeland, The Netherlands) to minimize reflection during intravital microscopic imaging. The optical chamber was horizontally secured onto the heated stage and the stage was positioned under the microscope on a Z-axis lab jack positioning stage. The exposed tissue was kept moist with sterile phosphate buffered saline (PBS, 37 °C) to deter desiccation and consequent twitching of cutaneous muscles.

### 2.8. Flow Cytometric Analysis of Hamster and Human Platelet Staining by Molecular Probes, Antibodies, and LUVETs

The staining of hamster and human platelets with 5(6)-carboxyfluorescein (CF), calcein, fluorescently labeled antibodies raised against P-selectin (CD62P; a platelet activation-dependent surface receptor [37]), and fluorophore-containing LUVETs was assayed by flow cytometry to confirm previously published results [15,16,23,24,38] in a different laboratory setting and to determine platelet–LUVET association with respect to the DPPC:DSPE-PEG (96:4) formulation.

LUVETs composed of DPPC:DSPE-PEG (96:4) and DSPC:DSPE-PEG (96:4) were prepared as described in Section 2.2 at a final phospholipid concentration of 5 mM. The lipid films were hydrated with an iso-osmolar 2-mM CF solution that had been prepared from a 34-mM stock solution in physiological buffer (Section 2.9). Directly before incubation with platelets, the LUVETs were subjected to 2 sequential steps of size exclusion chromatography (Section 2.2) to completely remove unencapsulated CF and were subsequently stored at 4 °C in the dark. A 30% LUVET retention rate during sequential gelfiltration was accounted for when reporting final phospholipid concentrations in biological samples. LUVETs were characterized (size, polydispersity index, zeta potential, Section 2.2) post-gelfiltration.

Whole blood was accessed from hamsters as described in Section 2.7 and collected into a 1-mL syringe (19 G needle) containing 100 µL of 3.8% (*w*/*v*) sodium citrate in PBS (1:9 citrate:blood volume ratio, 0.38% final citrate concentration). The blood was transferred to a 1.5-mL Eppendorf tube and centrifuged at 200 × g for 10 min at RT. Platelet-rich plasma (PRP) was carefully aspirated and diluted with PBS (RT) to 25 × 10^3^ platelets/µL following counting (AMC Department of Clinical Chemistry). Whole human blood was drawn from a healthy volunteer (MIvR) into a 15-mL polystyrene centrifuge tube (BD Biosciences) containing 3.8% (*w*/*v*) sodium citrate in PBS in a 1:9 volume ratio to blood using an open collection system. PRP was obtained and processed as described for hamster blood.

Diluted PRP (40 µL) was incubated with 1 µL of 10 µg/mL convulxin for 10 min at RT (activated platelets) or without convulxin (resting platelets). Subsequently, 10 µL of the gelfiltered LUVET suspension was added to the diluted PRP to an fc of 0.70 mM lipid (corresponding to a platelet:lipid ratio of 2 × 10^13^ platelets/mole LUVET lipids) and incubated for 30 min at RT in the dark (N = 3–4 independent experiments). Alternatively, 40 µL of PRP was incubated with 10 µL of CF (1000 × total dilution, 34 µM fc) and calcein (1000× total dilution, 50 µM fc, Section 2.5) as well as 2 μL of anti-CD62P-FITC antibodies (500 μg/mL) or isotype control antibodies (anti-IgG_1K_-FITC, 500 μg/mL) for 30 min at RT in the dark (N = 2 independent experiments). Platelets were washed by addition of 0.5 mL of PBS (RT) and centrifugation for 5 min at 288× *g* at RT. The supernatant was decanted to remove unassociated LUVETs/fluorophores/antibodies. Finally, 0.5 mL PBS (4 °C) was added before flow cytometry (EPICS XL-MCL, Beckman Coulter, Fullerton, CA, USA). Ten thousand events were collected in the platelet gate.

Data analysis and plotting were performed in FlowJo software (BD Biosciences). Total fluorescence was calculated by multiplying the mean fluorescence by the total cell count in the gated region. The fold-increase in the fluorescence of platelets that had been incubated with LUVETs/fluorophores/antibodies was calculated relative to the total fluorescence of unstained platelets. Data were presented as mean ± SD (N ≥ 3) or variance (for N = 2).

### 2.9. Preparation of Fluorophore-, Antibody-, and Liposome-Containing Solutions for Intravenous Administration into Hamsters

The iso-osmolar calcein solution (49.6 mM, Section 2.5) was used without further dilution and infused at 40 µL/animal, equating to 1.9 µmol per 100 g body weight and a blood concentration of 254 µM calcein at an average hamster blood volume of 78 mL/kg. Calcein exhibits absorption and fluorescence emission maxima at 494 and 517 nm, respectively, in physiological buffer (20 µM calcein), measured with a Lambda 18 spectrophotometer (Perkin Elmer, Wellesley, MA, USA) and an SPF 500C spectrofluorometer (American Instrument Company).

A 34-mM CF solution was prepared in a similar manner as the calcein solution (Section 2.5). The CF solution was diluted to 3.4 mM with sterile PBS and injected at a volume of 150 µL/hamster, corresponding to 510 nmol per 100 g body weight and a blood concentration of 65 µM CF. CF exhibits absorption and fluorescence emission maxima at 492 and 520 nm, respectively, in physiological buffer (20 µM CF).

FITC-conjugated rat anti-mouse CD62P (P-selectin) monoclonal antibodies (0.5 mg/mL) were infused at a volume of 150 µL/hamster without further dilution.

CF-encapsulating DSPC:DSPE-PEG (96:4) LUVETs were prepared and gelfiltered as described in Section 2.2, with the exception that the phospholipid films were hydrated with physiological buffer containing 20 µM CF to a 10-mM final phospholipid concentration. These ‘non-thermosensitive’ LUVETs, where only the acyl chains of the main component phospholipids differed by 2 carbons in length compared to the thermosensitive variant (DPPC substituted by DSPC), were prepared to prevent CF leakage at body temperature (Figure S7) and were infused at 200 and 400 µL/hamster, accounting for a systemic phospholipid concentration of approximately 250 and 500 µM, respectively. The concentrations are in line with systemic phospholipid concentrations used in rat and mouse models [23]. The fluorescence excitation and emission spectra of liposomal CF did not notably differ from the absorption and fluorescence emission spectra of unencapsulated CF (data not shown).

Finally, DPPC:NBD-PC:DSPE-PEG (91:5:4) LUVETs were prepared as described in Section 2.2, with the exception that the phospholipid films were hydrated with physiological buffer and no size exclusion chromatography was necessary. The fluorophore NBD was covalently bound to C_12_ of the *sn*2 acyl chain. The LUVETs were infused at a volume of 400 µL/hamster, accounting for a systemic phospholipid concentration of approximately 500 µM. The absorption and fluorescence emission maximum of NBD is 464 and 531 nm, respectively, in chloroform (i.e., lipophilic environment).

The fluorophore-containing solutions, antibodies, and NBD-labeled LUVETs were heated to body temperature on the heated stage before infusion and shielded from light, while the CF-encapsulating LUVETs were kept on ice in the dark.

### 2.10. Intravital Fluorescence Microscopy and Laser-Induced Thrombosis Model

The microscopy setup, which had been previously used for fluorescence-based intravital imaging and spectroscopy [39,40], consisted of a custom-modified stereo fluorescence microscope (M165FC, Leica, Wetzlar, Germany) equipped with a 1× Planapo objective (Leica), a Peltier-cooled DFC420C color camera (Leica) attached to a 0.5× C-mount, and an EL6000 light source (Leica). The spectral output of the light source was measured with a QE65000 spectrometer (Ocean Optics, Largo, FL, USA) integrated into the microscope optical path and is presented in Figure S4A. The light source and fluorescence filter set (filter set model B, 507-nm longpass emission filter, Leica, Figure S4B), of which the excitation and emission filter bandwidths were also measured, were attuned to the spectral properties of calcein, CF, FITC, and NBD-PC.

Intravital imaging was enabled by reconfiguring the camera settings to multi-time acquisition in LAS (control software, Leica). The hardware and software settings are summarized in Table S2. The camera was white-balanced against a plain sheet of white paper before imaging to remove unrealistic color casts. Automated shutter control of the excitation light source was engaged to ensure 1-ms illumination for fluorophore excitation and subsequent closing of the EL6000 shutter to prevent fluorophore bleaching between measurements. In some experiments, an external light source (model Short arc 270 plus, Karl Storz, Tuttlingen, Germany, Figure S4C) was used for transillumination. The light was delivered to the optical chamber via an SMA-coupled, 1 mm multimode fiber optic (Thorlabs, Newton, NJ, USA) and reflected through the dorsal skin. The fiber optic was connected to the Storz light source via a customized adapter. Imaging was performed under dim light conditions.

For the induction of thrombi, an external laser system was custom-built by the Department of Medical Innovation and Development (BCS) (Supplementary Section S2.5 and Figure S5). A 532-nm diode-pumped solid state laser with manual power modulation (0–1500 mW) was purchased from CNI Laser (model MGL-FN-532, Changchun, China) (Figure S4D). The laser was mounted on an XY translator stage and steel base for stability. Pulse width was controlled by a high-speed shutter (model Lambda SC SmartShutter, Sutter Instrument, Novato, CA, USA). The focal plane of the microscope and the laser were fixed along the Z-axis so that the target blood vessel was maneuvered into the joint focal plane by manually sliding the heated stage (lateral) and adjusting the lab jack (height).

Thrombosis was induced as described previously [5,6,15] with single or multiple laser pulses at 224 mW output power (FieldMate power meter, Coherent, Santa Clara, CA, USA), a spot size of 2 × 10^−3^ mm^2^, pulse widths ranging from 1 to 180 ms, and an incident radiant exposure of 10–1734 J/cm^2^. The number of laser pulses and pulse width used are indicated per experiment in Table S2.

The following groups were studied in this pilot experiment: (1) a negative control group (N = 4) where thrombi were induced in the absence of any injected fluorophores; (2) a positive control that was administered anti-CD62P monoclonal antibodies (N = 1; Section 2.9); (3) a positive control that was administered calcein (N = 1; Section 2.9); (4) a positive control that was administered CF (N = 1; Section 2.9); (5) a group that was administered CF-encapsulating DSPC:DSPE-PEG (96:4) LUVETs (N = 2; Section 2.9); and (6) a hamster that was administered DPPC:NBD-PC:DSPE-PEG (91:5:4) LUVETs (N = 1; Section 2.9). At the end of the experiment, animals were sacrificed by exsanguination via cardiac puncture and cervical dislocation. The urine was collected from the bladder and organs were extracted, trimmed (fascia and fat), rinsed with PBS, dried with a paper towel, and imaged in fluorescence mode (B filter set).

### 2.11. Image Analysis

The multi-time acquisition feature in LAS software generates a sequence of stills captured at a predefined time interval (Table S2). The unedited stills were imported into Premiere Pro (Adobe, San Jose, CA, USA) and rendered into AVI movie files at 25 fps, encompassing a 36.9–87.6-fold acceleration of the actual image acquisition rate, without any further editing. The frames with the most profound thrombus (group 1) and most intense thrombus fluorescence (groups 2–6) were isolated and imported into Photoshop (Adobe). The isolated frame of each group was duplicated. The original frame displayed the unfiltered true colors at wavelengths ≥ 507 nm, while the duplicate frame was filtered for the green hue range (496–559 nm; Figure S6). Given the fact that the camera was white-balanced and therefore captured true colors in the region of interest, the green hue range corresponds to the fluorescence emission range of all used fluorophores (Figure S4). The software-filtered duplicates therefore show whether any of the fluorophores were entrapped in the laser-induced thrombus.

## 3. Results

### 3.1. Tranexamic Acid-Encapsulating DPPC:DSPE-PEG Liposomes Have the Most Favorable Physicochemical Properties for Antifibrinolytic SSPLT

**Figure 2:**
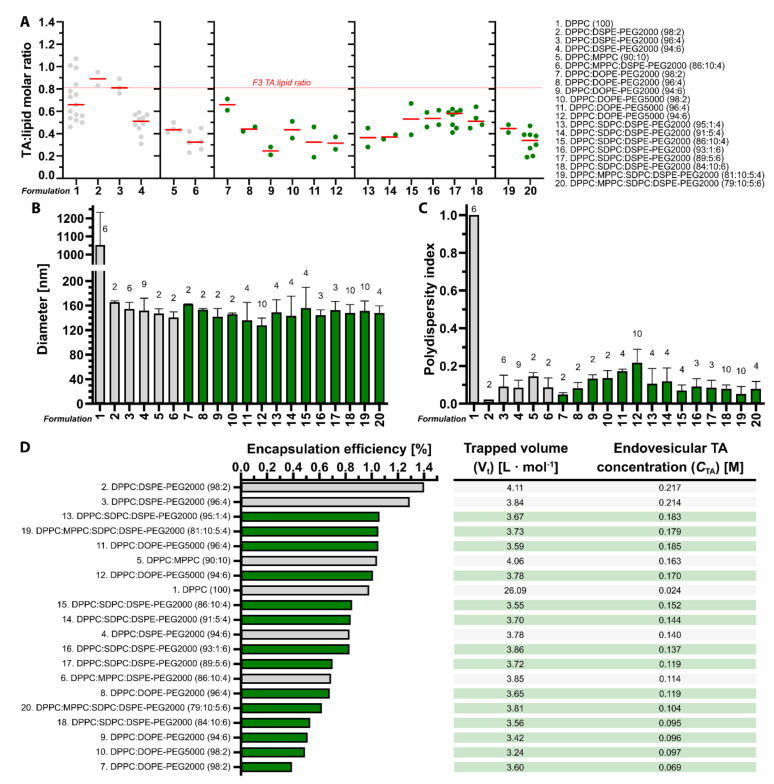
Physicochemical properties of tranexamic acid (TA)-encapsulating liposomal formulations. (**A**) TA:lipid molar ratio plotted per formulation. Dots represent individual experiments and short red lines represent the mean. The protracted red line, corresponding to the mean TA:lipid ratio of DPPC:DSPE-PEG liposomes—the prime candidate formulation for antifibrinolytic SSPLT—was inserted for purposes of comparison. The mean ± SD diameter (**B**) and polydispersity index (**C**) were measured by photon correlation spectroscopy. In (**D**), the encapsulation efficiency was calculated from the measured TA:lipid ratio after size exclusion chromatography in relation to the initial input of TA and lipids. The trapped volume was extrapolated from the mean liposomal diameter as described in Section 2.2. Data in gray have been published previously [16] and were included for purposes of reference and comparison.

In an attritional approach aimed at bringing antifibrinolytic SSPLT to the clinical setting, several parameters must be accounted for early in the development stage. The first is the amount of intraliposomal TA molecules that can be released during a heat stimulus, which is dictated by the TA:lipid ratio and *C*_TA_. The goal is to formulate thermosensitive liposomes with maximum TA encapsulation so that a heat-induced antifibrinolytic environment can be realized in PWS blood vessels with a minimum number of locally accumulated liposomes. In our preceding study [16], two of six liposomal formulations were deemed candidates for antifibrinolytic SSPLT based on their physicochemical properties and release kinetics in buffered solution. These formulations are DPPC:DSPE-PEG (96:4) and DPPC:MPPC:DSPE-PEG (86:10:4); i.e., formulations 3 and 6 in Figure 2. DPPC:DSPE-PEG liposomes are sufficiently PEGylated [41,42] and properly sized [28,43] to prevent rapid clearance from the circulation, have a relatively high TA:lipid ratio and E_eff_ that translate to a favorable *C*_TA_, and release nearly all TA cargo during a short period of exposure to mild hyperthermia but not near body temperature (37.0 °C) [16]. The MPPC-containing variant represents a formulation with accelerated release kinetics at the expense of *C*_TA_, serving as a back-up formulation for the DPPC:DSPE-PEG liposomes in case these do not pass attrition.

In an attempt to further increase TA:lipid ratio and *C*_TA_ while upholding the thermosensitive character of the liposomes, 14 additional formulations were prepared and benchmarked against the two previously established candidate formulations. In addition to extending the number of possible back-up formulations, the efforts also anticipated on the implementation of alternative triggered release mechanisms for SSPLT, including the perturbation of membrane stability by the photochemical modification of mono- and poly-alkenes in unsaturated acyl chains [14].

Accordingly, DSPE-PEG was substituted by DOPE-PEG2000 and DOPE-PEG5000 (formulations 7–12, Figure 2), which contain a Δ9-cis alkene in each acyl chain that is susceptible to peroxidation by photodynamically produced singlet oxygen [44]. The DOPE-PEG5000 was investigated because of its longer circulation half-life than DOPE-PEG2000 [43,45]. As shown in Figure 2A, the TA:lipid ratios were lower in liposomes sterically stabilized with DOPE-PEG5000 compared to DOPE-PEG2000 and declined with increased PEGylation in both classes, most likely due to the spatial occupation of the aqueous compartment by PEG. At comparable particle sizes in the range of 128–163 nm (Figure 2B), relatively homogenous size distribution (Figure 2C), and E_eff_ ranging from 0.39–1.04%, the *C*_TA_s of DOPE-PEG formulations stretched from 69 to 163 mM (Figure 2D). These properties are inferior to those of DPPC:DSPE-PEG (96:4) liposomes but comparable to those of DPPC:MPPC:DSPE-PEG (86:10:4) liposomes.

Next, the physicochemical properties of liposomes containing poly-unsaturated acyl chains were investigated, which entailed the inclusion of SDPC (Δ4,7,10,13,16,19-cis alkenes in the *sn*2 acyl chain) up to 10 mol % at the expense of DPPC (formulations 13–18, Figure 2). SDPC offers numerous oxidation sites while maintaining low membrane permeability in native state [46]. The inclusion of SDPC was associated with a reduction in TA:lipid ratio compared to DPPC-only formulations containing an equimolar fraction of DSPE-PEG. The inclusion of 6 mol % DSPE-PEG did not reduce the TA:lipid lipid ratio versus 4 mol % DSPE-PEG, which was previously observed in SDPC-lacking liposomes [16]. The TA:lipid ratio was not notably influenced by the molar fraction of SDPC (Figure 2A). The particle sizes were comparable to the SDPC-lacking liposomes (Figure 2B) and equally monodisperse (Figure 2C), yielding *C*_TA_s in the range of 137–185 mM. The higher *C*_TA_s were mainly associated with liposomes containing 6 mol % DSPE-PEG. Based on these properties, the SDPC liposomes are favorable over DOPE-PEG liposomes if photochemical triggering is chosen as a complementary release mechanism.

Finally, two SDPC (5 mol %) formulations were prepared that also contained 10 mol % MPPC at the expense of DPPC and either 4 or 6 mol % DSPE-PEG (formulations 19 and 20, Figure 2). Inclusion of MPPC into SDPC liposomes containing 4 mol % of DSPE-PEG increased the TA:lipid ratio from 0.37 to 0.45 and the C_TA_ from 144 to 179 mM without imparting significant deleterious differences in liposome size (Figure 2B), polydispersity index (Figure 2C), and E_eff_ (Figure 2D). Conversely, the presence of MPPC in SDPC liposomes containing 6 mol % DSPE-PEG reduced the TA:lipid ratio from 0.54 to 0.32 and the C_TA_ from 119 to 104 mM (Figure 2A or Figure 2D) without markedly affecting the other physicochemical properties (Figure 2B–D) compared to MPPC-lacking SDPC liposomes.

In conclusion, none of the newly tested formulations offered additional benefits over the prime candidate formulation composed of DPPC:DSPE-PEG (96:4; *F3*) in terms of TA:lipid ratio, E_eff_, and *C*_TA_. The TA E_eff_s are low for all formulations compared to other liposomal encapsulants (Table S3 [47,48,49,50,51,52,53,54,55,56,57,58,59,60,61,62,63,64,65,66]), which was expected as explained in Supplementary Section S3.1. Further experiments therefore remained focused on the DPPC:DSPE-PEG (96:4) and DPPC:MPPC:DSPE-PEG (86:10:4) LUVETs. Nevertheless, PEGylated SDPC-containing formulations could be reverted to for antifibrinolytic SSPLT if the DPPC:(MPPC:)DSPE-PEG LUVETs fail in animal studies or if photochemical triggering mechanisms are chosen over thermodynamic triggering.

### 3.2. Tranexamic Acid-Encapsulating PEGylated Thermosensitive Liposomes Release Content at Temperatures Equal to and Above the Phase Transition Temperature in Buffered Solution

The second criterion to be fulfilled is that the thermosensitive liposomes release as much cargo as possible in the shortest time frame. The clinical endpoint is the complete occlusion of the blood vessel, which is easier to achieve during the growth phase of the thrombus (i.e., during the first 10 min after laser illumination [15]) than during the breakdown phase (after 10 min post-lasing [15]). At T ≈ T_m_, DPPC:DSPE-PEG (96:4) LUVETs release 95.5% of TA during 2.5 min of equilibrated heating at a release rate of 0.78%·s^−1^, whereas DPPC:MPPC:DSPE-PEG (86:10:4) LUVETs release 93.0% of TA during 2.0 min of equilibrated heating at a release rate of 1.02%·s^−1^ [16]. Inasmuch as maintaining a constant and exact intravascular temperature in patients is not feasible, local temperature gyrations upward of T_m_ will likely occur during skin heating. Consequently, TA release at T ≈ T_m_ as well as at T > T_m_ would be desirable. Currently, it is unknown whether TA release occurs in the L_α_ phase of DPPC bilayers, as has been reported for other molecules [67,68]. TA release from DPPC:DSPE-PEG (96:4) and DPPC:MPPC:DSPE-PEG (86:10:4) LUVETs was therefore investigated at T > T_m_ during 5 min of heating, i.e., in line with the thrombus dynamics [15] and previously established complete release times [16]. Despite acceptable TA:lipid ratios and *C*_TA_s, the additional candidate formulations DPPC:SDPC:DSPE-PEG (91:5:4) and DPPC:MPPC:SDPC:DSPE-PEG (81:10:5:4) (Section 3.1) were not included in the analysis because the cold shoulder in the DSC thermogram extended into the body temperature range for both formulations (Figure S7) and may cause TA release under normothermic conditions.

**Figure 3:**
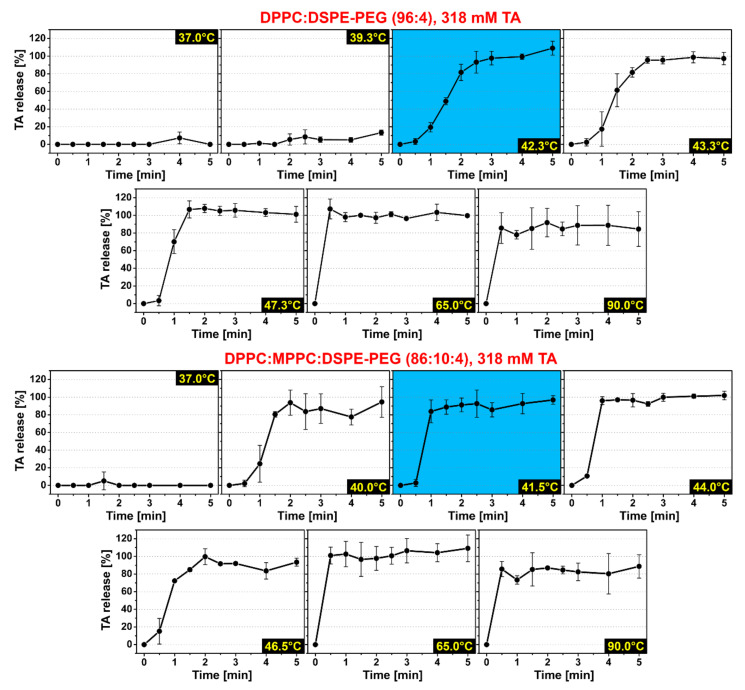
Heat-induced tranexamic acid (TA) release from DPPC:DSPE-PEG (96:4) large unilamellar vesicles prepared by the extrusion technique (LUVETs) (top) and DPPC:MPPC:DSPE-PEG (86:10:4) LUVETs (bottom) plotted as a function of heating time at varying temperatures from 37.0 to 90.0 °C. Temperatures are indicated in the black labels. The panels in blue represent heating at T = T_m_ as measured by differential scanning calorimetry (Figure S7). Released TA concentration is expressed as a mean ± SD percentage of total liposomal TA content (N = 3–6 per time point from independent experiments conducted by 3 researchers). TA release from DPPC:MPPC:DSPE-PEG (86:10:4) LUVETs at 36 °C [16] is not shown because the data furnish no additional value to the release kinetics presented at 37 °C. The TA release kinetics from DPPC:DSPE-PEG (96:4) LUVETs at 39.3 and 42.3 °C and the TA release kinetics from DPPC:MPPC:DSPE-PEG (86:10:4) LUVETs at 41.5 °C were adapted from [16] and presented here for purposes of comparison.

As shown in Figure 3, heating of liposomes above the T_m_ did not perturb TA release but accelerated it. At temperatures up to T_m_ (42.2 °C; Figure S7), DPPC:DSPE-PEG (96:4) LUVETs released 0.0 ± 0.0%, 8.5 ± 8.0%, and 93.2 ± 12.3% of TA at 37.0, 39.3, and 42.3 °C, respectively, equating to a release rate of 0.00, 0.03, and 0.53%·s^−1^, respectively, during the first 3.0 min of heating. At temperatures above T_m_, DPPC:DSPE-PEG (96:4) LUVETs released 95.5 ± 3.6%, 106.7 ± 9.6%, 107.3 ± 11.3%, and 85.6 ± 17.4% of TA at 43.3, 47.3, 65.0, and 90.0 °C, respectively, equating to a release rate of 0.64 (plateau within 2.5 min), 1.19 (1.5 min), 3.58 (0.5 min), and 2.85%·s^−1^ (0.5 min), respectively.

At temperatures up to T_m_ (41.6 °C; Figure S7), DPPC:MPPC:DSPE-PEG (86:10:4) LUVETs released 0.0 ± 0.0%, 5.1 ± 10.1%, 80.5 ± 3.1%, and 88.8 ± 8.2% of TA at 36.0, 37.0, 40.0, and 41.5 °C, respectively, equating to a release rate of 0.00, 0.06, 0.89, and 0.99%·s^−1^, respectively, during the first 2.5 min of heating. At temperatures above T_m_, DPPC:MPPC:DSPE-PEG (86:10:4) LUVETs released 96.0 ± 4.4%, 72.3 ± 0.6%, 101.0 ± 9.6%, and 85.6 ± 8.7% of TA at 44.0, 46.5, 65.0, and 90.0 °C, respectively, equating to a release rate of 1.60 (plateau within 1.0 min), 1.21 (1.0 min), 3.37 (0.5 min), and 2.85%·s^−1^ (0.5 min), respectively.

In sum, DPPC:DSPE-PEG (96:4) LUVETs rapidly release TA at T_m_. TA release was accelerated at T > T_m_, i.e., in the L_α_ phase, where leakage of small molecules was not expected to occur extensively due to reorganization of phospholipid acyl chains to a more tightly packed conformation compared to T_m_ [69,70,71,72,73]. These findings are beneficial for antifibrinolytic SSPLT because the mild hyperthermia will not have to be confined to a narrow temperature range to achieve substantial TA release, making the clinical procedure easier.

### 3.3. Plasma Stabilizes Thermosensitive Liposomes and Reduces the Extent of Leakage at Body Temperature

Because membrane stability is known to be influenced by interactions with plasma components and individual proteins [67,74,75,76], fluorescence assays were performed with liposome-encapsulating calcein—acting as a TA mimetic—to determine passive leakage rates at body temperature from thermosensitive LUVETs in the presence of increasing PPP concentrations. Calcein was chosen because, unlike the more frequently used fluorophore CF [77], its fluorescence is not markedly quenched by plasma components [23]. The DPPC:DSPE-PEG (96:4) and DPPC:MPPC:DSPE-PEG (86:10:4) LUVETs, prepared with an iso-osmolar calcein solution relative to physiological buffer and plasma, had a mean ± SD diameter of 111 ± 2 and 130 ± 2 nm, respectively, and a mean ± SD polydispersity index of 0.282 ± 0.050 and 0.227 ± 0.023, respectively.

**Figure 4:**
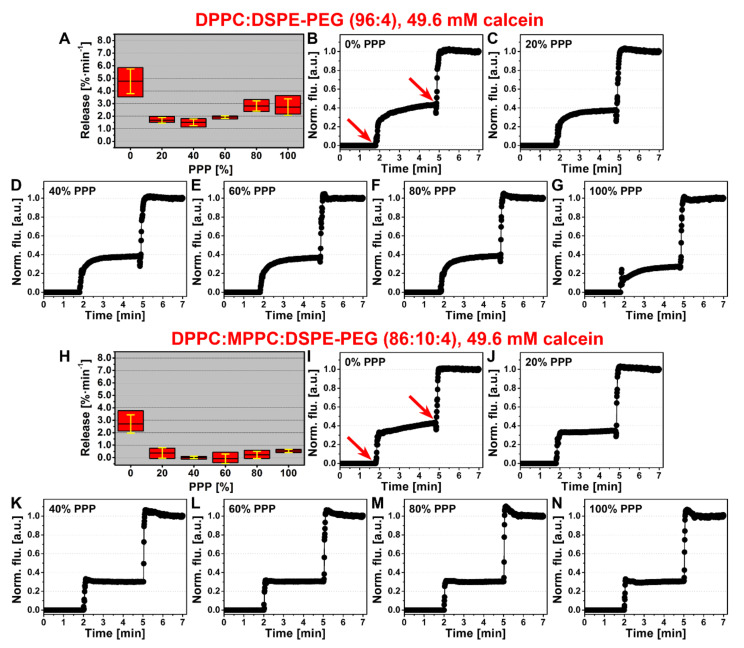
Calcein leakage at body temperature (37 °C) from DPPC:DSPE-PEG (96:4) LUVETs (**A**–**G**) and DPPC:MPPC:DSPE-PEG (86:10:4) LUVETs (H-N) that had been loaded with a self-quenched, iso-osmolar calcein solution relative to physiological buffer and plasma (0.293 osmol·kg^−1^). Leakage rates were plotted as a function of platelet-poor plasma (PPP) concentration (**A**,**H**) in modified box-whisker plots showing the maximum, minimum, mean, and SD release rate (N = 4 or 5 per group). Representative leakage kinetics are plotted vs. time at increasing PPP concentrations, shown in the upper left corner (**B**–**G**,**I**–**N**). The left red arrow in (**B**,**I**) indicates the time point at which LUVETs (15 µL) were added to the cuvette containing 1485 µL of physiological buffer and/or PPP, resulting in an instant increase in fluorescence due to residual fluorescence from incompletely quenched liposomal calcein, while the right red arrow indicates the addition of TX100 to induce 100% calcein release and unquenched fluorescence.

PPP imparted a stabilizing effect on passive calcein leakage rates in DPPC:DSPE-PEG (96:4) LUVETs and DPPC:MPPC:DSPE-PEG (86:10:4) LUVETs compared to physiological buffer at 37 °C (Figure 4). For DPPC:DSPE-PEG LUVETs the maximum and minimum mean reduction in leakage rate was observed at 40% PPP (1.51 ± 0.27%·min^−1^) and 80% PPP (2.81 ± 0.41%·min^−1^), respectively, corresponding to a reduction in the degree of leakage of 68% and 41% vs. physiological buffer, respectively. For DPPC:MPPC:DSPE-PEG LUVETs, the maximum and minimum mean reduction in leakage rate was observed at 100% PPP (0.53 ± 0.13%·min^−1^) and 60% PPP (−0.09 ± 0.38%·min^−1^), respectively, corresponding to a reduction in the degree of leakage of 80% and 100% vs. physiological buffer, respectively.

The experiments in physiological buffer and in 100% PPP were repeated at 4 °C and resulted in complete abrogation of calcein leakage (Figure S8), indicating that the temperature component is the main factor responsible for liposomal membrane permeability to calcein. Furthermore, the experiments were conducted at 37 °C with LUVETs that contained a hypo-osmolar calcein solution (0.210 osmol·kg^−1^) in the aqueous core. For DPPC:DSPE-PEG (96:4) LUVETs, the osmotic gradient reduced calcein leakage in physiological buffer by approximately 2.5-fold but had no effect on leakage rates in the presence of incremental fractions of PPP (Figure S9). The leakage rates in the plasma-containing groups were comparable to the leakage rates of respective LUVETs containing an iso-osmolar calcein solution in the aqueous compartment (Figure 4).

In contrast, DPPC:MPPC:DSPE-PEG (86:10:4) LUVETs became highly permeable to calcein, exhibiting immediate and complete leakage of calcein in physiological buffer and 4.53–5.95%·min^−1^ release rates in the presence of PPP (i.e., ~10-fold greater compared to iso-osmolar LUVETs, Figure 4), which proceeded in a PPP-independent manner (Figure S9). Moreover, the difference of 0.083 osmol·kg^−1^ shifted the onset of content leakage to temperatures of > 20 °C below T_m_ (i.e., around 20 °C) (Figure S10), underscoring the necessity of osmotic balance in regard to MPPC-containing thermosensitive liposomes intended for systemic administration.

Taken together, the presence of plasma reduces the leakage of liposomal content at body temperature, which is ideal for in vivo and clinical application of antifibrinolytic SSPLT. The passive release of high quantities of TA into the systemic circulation of non-coagulopathic patients could be considered a contra-indication insofar as TA could cause thrombotic complications that otherwise would not manifest. Osmotic imbalances are inconsequential for DPPC:DSPE-PEG (96:4) LUVETs but should be avoided in DPPC:MPPC:DSPE-PEG (86:10:4) LUVETs to prevent non-induced leakage.

### 3.4. LUVETs Associate with Hamster and Human Platelets

Platelet labeling with molecular probes and antibodies and association with LUVETs was investigated by flow cytometry as a prelude to the in vivo study and in anticipation of future clinical implementation of antifibrinolytic SSPLT. Platelets could be labeled with CF and calcein, which occurred irrespective of activation status and species (hamster and human) at approximately equal intensity (Figure S11A–F). In contrast, convulxin-activated hamster and human platelets were bound by anti-CD62P-FITC antibodies, whereas resting platelets were not. The antibody binding to activated platelets was selective as evidenced by the absence of fluorescent platelet labeling with anti-IgG1K-FITC antibody isotype controls (Figure S11G,H). Based on these data, the molecular probes and antibodies could be used to render laser-induced thrombi fluorescent in vivo.

**Figure 5:**
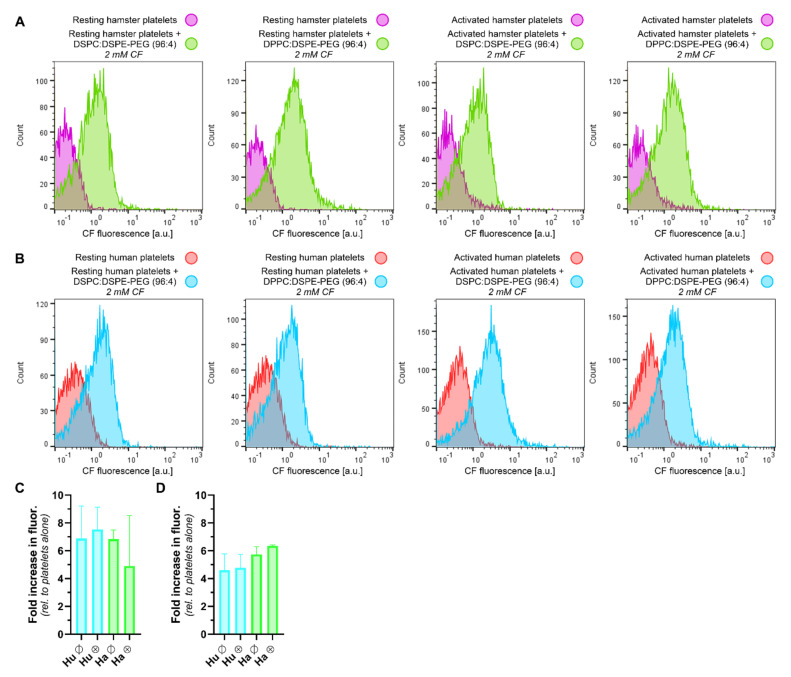
Representative flow cytograms of resting and convulxin-activated hamster (**A**) and human (**B**) platelets that had been incubated with 5(6)-carboxyfluorescein (CF)-encapsulating DSPC:DSPE-PEG (96:4) and DPPC:DSPE-PEG (96:4) LUVETs (N = 3–4 per group). The purple (**A**) and red traces (**B**) represent fluorescence histograms of platelets that had not been incubated with LUVETs (control). The green (**A**) and blue traces (**B**) represent fluorescence histograms of platelets that had been incubated with LUVETs. The fold increase in fluorescence relative to non-labeled platelets is provided in (**C**) for DSPC:DSPE-PEG (96:4) LUVETs and (**D**) for DPPC:DSPE-PEG (96:4) LUVETs (Hu, human; Ha, hamster; ∅, resting platelets; ⊕, activated platelets).

Association between platelets and LUVETs would provide a valid reason to investigate LUVET incorporation into laser-induced thrombi in vivo without further modification of LUVET surface properties. Resting and convulxin-activated hamster and human platelets were therefore incubated for 30 min with CF-encapsulating DSPC:DSPE-PEG LUVETs (96:4, 116.2 ± 4.7 nm diameter, polydispersity index of 0.183 ± 0.023, zeta potential of −1.5 ± 3.4 mV) and DPPC:DSPE-PEG LUVETs (96:4, 116.0 ± 5.6 nm diameter, polydispersity index of 0.383 ± 0.010, zeta potential of −1.6 ± 3.2 mV) and analyzed flow cytometrically. As shown in Figure 5, LUVETs associated with platelets in a platelet activation-, LUVET composition-, and species-independent manner at near-equal magnitude. The results yield credence to the hypothesis that these non-(immuno)targeted liposomes could be used to deliver antifibrinolytics to laser-induced thrombi, which was investigated next.

### 3.5. LUVETs Do Not Incorporate into Laser-Induced Thrombi In Vivo

The ability of PEGylated liposomes to incorporate into thrombi without active (immune)targeting to clot constituents such as activated platelets or fibrin [14] was explored in an established hamster model for studying endovascular laser-tissue interactions in PWS vascular analogues [5,6,15]. The in vivo study was predicated on the low number of TA-carrying thermosensitive liposomes that are required to create a potent antifibrinolytic milieu in a 500-µm long vessel segment [16,78]. In that respect, passive accumulation of PEGylated LUVETs may be sufficient for antifibrinolytic SSPLT with TA as encapsulant. Dorsal venules were laser-illuminated and intravitally imaged following infusion of fluorescent platelet-staining antibodies and molecules [15,38] as positive controls (Section 3.4) and fluorescent phosphatidylcholine-DSPE-PEG (96:4) LUVETs. The LUVETs represent the prime thermosensitive candidate formulation for antifibrinolytic SSPLT (Figure 2, *F3*), as established previously [16] and confirmed in this study. The DSPC:DSPE-PEG (96:4) LUVETs contained CF in the aqueous compartment, while the DPPC:NBD-PC:DSPE-PEG (91:5:4) LUVETs contained a molar fraction of fluorescently labeled lipid. Images were analyzed offline for intrathrombus green fluorescence to gauge the extent of molecular labeling and LUVET incorporation.

**Figure 6:**
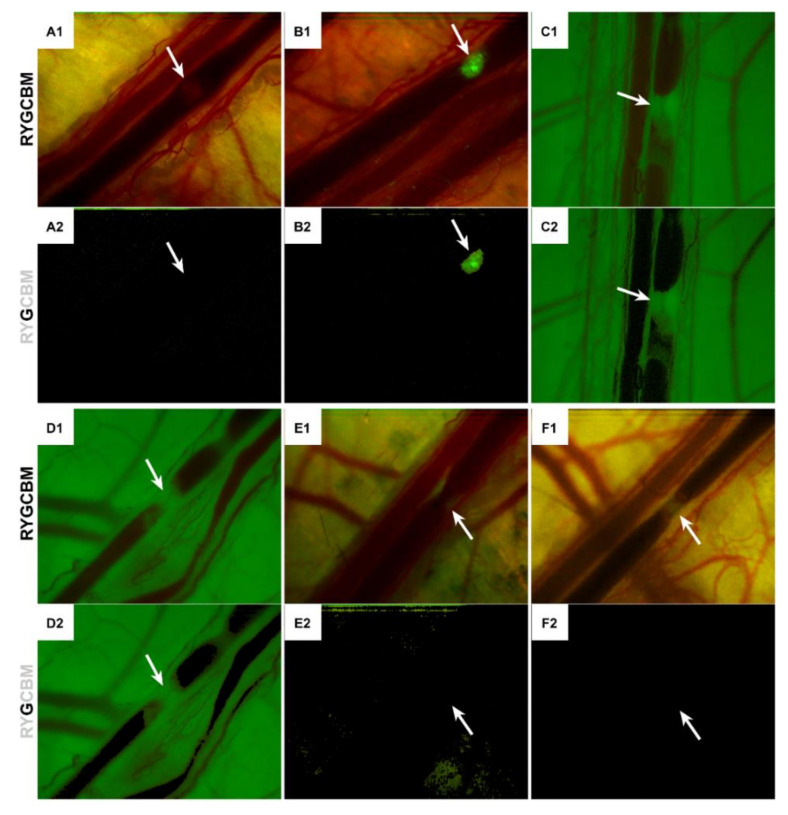
Intravital fluorescence imaging of laser-induced thrombi in hamster dorsal venules. (**A**) Negative control (no infusion of fluorophores); (**B**) positive control (FITC-labeled anti-CD62P monoclonal antibodies, 150 µL of 0.5 mg/mL); (**C**) calcein (40 µL of 49.6 mM); (**D**) CF (150 µL of 3.4 mM); (**E**) CF-encapsulating DSPC:DSPE-PEG (96:4) LUVETs (400 µL of 10 mM phospholipids); and (**F**) DPPC:NBD-PC:DSPE-PEG (91:5:4) LUVETs (400 µL of 10 mM phospholipids). The top panels designated as ‘1’ represent the unedited frames with the vastest (**A**) and most intensely fluorescent thrombus (**B**–**F**) of the entire video sequence. All hues (Red, Yellow, Green, Cyan, Blue, and Magenta, left of panels) are included and unfiltered. The bottom panels designated as ‘2’ are the green-filtered duplicates (RYGCBM) intended to show the presence of fluorophores in the thrombus (arrow). Thrombi are only green-fluorescent in the positive control group (**B**) and the free fluorophore groups (**C**,**D**), indicating that the LUVETs were not sufficiently entrapped in laser-induced thrombi.

Laser illumination resulted in thermal coagulum formation and subsequent thrombosis, in line with what has been reported previously [5,6,15]. In the negative control group, where no fluorescent compounds had been infused, thrombi were discernible by dynamic vague patches at the site of laser-induced damage (Figure 6A1 and Figure S12), stemming from the hemoglobin-lacking platelet/fibrin clots being contrasted against a hemoglobin-rich dark circulation. In the presence of FITC-conjugated anti-CD62P antibodies, the thrombi were highly green fluorescent (Figure 6B), allowing the thrombus formation kinetics to be observed in time (Figure S13). Green-filtering of the video frames featuring the most voluminous thrombus (negative control, Figure 6A2) and the most intensely fluorescent thrombus (anti-CD62P-FITC antibodies, Figure 6B2) evinced that the negative control group did not produce any background fluorescence in the green wavelength range (507–559 nm) and established proof-of-concept for the imaging setup and image analysis, respectively. The antibody labeling of activated platelets was corroborated with calcein (Figure 6C) and CF (Figure 6D), both of which stain resting and activated platelets (Figure S11) [38]. Calcein and CF extravasated into perivascular tissue, given the green fluorescence of the dorsal skin. After ~90 min circulation, the anti-CD62P-FITC antibodies appeared to be present in the gallbladder and urine (Figure S14). After ~85 min circulation, CF was replete in the liver and gallbladder, stomach, intestines, and kidneys (Figure S15).

When PEGylated LUVETs were injected at a systemic phospholipid concentration of 0.5 mM, thrombi did form (Figure 6E1,F1), but no LUVET incorporation into laser-induced thrombi was observed for either formulation (Figure 6E2,F2). The LUVETs could be imaged by the microscope (Figure S16A and Figure S17A) and were retrieved in the gallbladder and urine (Figure S16D,J or Figure S17D,J), so the absence of intrathrombus accumulation was not attributable to technical details. It is therefore concluded that, despite platelet-LUVET interactions in vitro (Figure 5), the LUVETs do not sufficiently incorporate into laser-induced thrombi to warrant their use in antifibrinolytic SSPLT without liposome-conjugated thrombus-targeting ligands such as antibodies or antibody derivatives (Fab’ fragments or nanobodies against CD62P) [14,15].

## 4. Discussion

The main aim of this second study on antifibrinolytic SSPLT was to investigate the potential utility of PEGylated thermosensitive LUVETs in populating a developing laser-induced thrombus, which is a prerequisite for creating a local antifibrinolytic environment following exposure of a laser-treated PWS to mild hyperthermia and consequent heat-triggered TA release (Figure 1). The first study on anti-fibrinolytic SSPLT [16] confirmed that the thermosensitive formulations were stable at body temperature and released TA upon a heat stimulus at a rate that aligns very well with laser-induced thrombus dynamics [15] and with the envisaged treatment protocol in a clinical setting. These confirmatory results paved the way to the present study, where several developmental steps needed to be addressed before proceeding to in vivo proof-of-concept studies.

First, the number of TA-encapsulating candidate formulations was expanded to ascertain the most optimal formulation while taking into account alternative triggered release mechanisms (thermal and photochemical). In that respect, we developed a number of comparable liposomal formulations containing the lipophilic photosensitizer zinc phthalocyanine [79,80,81,82] for photodynamic therapy and demonstrated their potential to oxidize biomolecules and molecular probes upon illumination [27,39,83]. The singlet oxygen produced by light-activated zinc phthalocyanine, which exhibits no dark toxicity [80], could equally be exploited to oxidize alkenes [84,85,86], such as in DOPE-PEG and SDPC co-formulated into thermosensitive liposomal membranes, and induce TA release as a result of membrane oxidation [14].

Fourteen novel thermosensive formulations were characterized and benchmarked for the most relevant parameters (TA:lipid ratio, E_eff_, and *C*_TA_), which resulted in the conclusion that DSPC:DSPE-PEG (96:4) and DPPC:MPPC:DSPE-PEG (86:10:4) LUVETs are the best-suited formulations for antifibrinolytic SSPLT when combined with thermally induced release. In addition to exhibiting a high TA:lipid ratio, E_eff_, and *C*_TA_, both formulations also carry physiologically justifiable phase transition properties relative to body temperature. The incorporation of mono-unsaturated and poly-unsaturated lipids into the liposomes shifted the T_m_ towards lower temperatures and hence introduced the risk of TA leakage following intravenous administration. DSPC:DSPE-PEG (96:4) and DPPC:MPPC:DSPE-PEG (86:10:4) LUVETs exhibited no-to-minimal TA release at 37 °C, which is the most desirable pharmacokinetic profile when dealing with this class of drugs being administered into non-coagulopathic patients. Another consideration in the attrition process was the simplicity and stability of the component phospholipids, which are important factors in later GMP production stages and drug pricing. The formulation is composed of cheap, commercially available saturated phospholipids that are inherently non-toxic [87,88], even when encapsulating TA [16], and therefore not susceptible to (per)oxidative modification [89] during storage.

Second, no information was available on TA release at T > T_m_. It is clinically difficult to achieve an equal temperature distribution across a tissue volume where the target vessels are situated up to a depth of 0.9 mm from the skin surface [90,91] and where semi-occluded blood vessels arguably account for temperature gyrations due to convection [13,92,93]. Accordingly, unhampered TA release from liposomes in the L_α_ phase is a desirable endpoint to make the second phase of SSPLT (heat-induced TA release, Figure 1) practically feasible in that heating would not have to occur in a narrow temperature range. The TA release assays evinced that release rates and temperature are governed by a directly proportional relationship, whereby near-complete TA release is achieved in 2.0–2.5 min at T_m_ that is accelerated at T > T_m_. The kinetics are also ideal for a clinical setting, as the laser-treated PWS can subsequently be illuminated with red light for the necessary time frame while the patient is recovering from the first part of the treatment regimen (photocoagulation with a yellow laser, Figure 1).

Thirdly, it was not known how the stability of the liposomes and retention of content would be affected by plasma at body temperature, which is an important factor when developing liposomal formulations destined for intravenous administration. Plasma, but also proteins, are known to alter liposomal membrane properties, including T_m_, and phospholipid bilayer stability, which could lead to content release before triggered release [67,74,75,76,94,95]. To test this, TA was substituted with calcein because the assay we had developed for TA quantification [16] would be impaired by amine-containing molecules such as plasma proteins. Moreover, the incorporation of calcein at self-quenching and iso-osmolar concentration allowed for real-time stability analysis. Spectrofluorometric measurements confirmed that plasma had a stabilizing impact on the LUVETs, that together with all previous results provided a ‘go-signal’ towards in vivo proof-of-concept.

Following in vitro confirmation that thermosensitive PEGylated LUVETs interact with platelets, we employed a hamster dorsal skinfold model [5,6,15] to gauge whether the LUVETs would sufficiently populate the developing laser-induced thrombus via passive incorporation. These experiments were performed based on previous calculations on the number of liposomes required to instill an antifibrinolytic effect in a 500-µm PWS blood vessel segment [16], which suggested that passive intrathrombus trapping of TA-loaded liposomes may be sufficient. Such a scenario would expedite the clinical translation of antifibrinolytic SSPLT because the need for conjugating thrombus-targeting ligands to liposomes would no longer be necessary, accounting for a simpler and more affordable photonanomedicine for PWS treatment. However, we found no LUVET incorporation into laser-induced thrombi in vivo, as evidenced by the absence of green fluorescence from CF in the DSPC:DSPE-PEG (96:4) LUVETs and NBD in the DPPC:NBD-PC:DSPE-PEG (91:5:4) LUVETs in the laser-induced thrombi. Both liposomal formulations could be imaged by the fluorescence microscope when presented as smears of the LUVET-containing solution and urine collected at the end of the experiment. The thrombi did not incorporate sufficient LUVETs, despite the fact that the LUVETs have been shown to associate with and/or to be taken up by all cellular constituents in the thrombus, namely platelets, leukocytes, and erythrocytes [16,23,24,96]. Moreover, the longest camera exposure times were used in the in vivo LUVET experiments to maximize the possibility that liposomal CF and NBD would be imaged, further mitigating the likelihood that LUVET fluorescence was not registered by the camera due to technical reasons.

In the final analysis, passive targeting of LUVETs to laser-induced thrombi will most likely not work in vivo, necessitating the implementation of active thrombus targeting strategies with respect to TA-based antifibrinolytic SSPLT. Numerous options to achieve such active targeting have been addressed in [14], which include antibodies, antibody derivatives, or peptides directed at preferably platelet activation-dependent surface proteins such as CD62P. Conjugation of these ligands to the distal end of PEG chains, as was performed in [82] by simple click chemistry, is not expected to alter the physicochemical properties of the phospholipid bilayer. Consequently, the release kinetics under a hyperthermic stimulus should be comparable to what was reported in this study following the coupling of targeting ligands to thermosensitive LUVETs.

## Supplementary Materials

The following are available online at https://www.mdpi.com/1999-4923/12/6/591/s1: Table S1. List of chemicals and reagents, Table S2. Summary of experimental settings used in the in vivo study, Figure S1. Osmolarities of the different solutions used for liposome and buffer preparation, plotted as a function of solute concentration, Table S3. Summary of physicochemical properties of liposomal formulations encapsulating different drugs, Figure S2. Experimental workflow related to the quantitative determination of heat-induced TA release from thermosensitive DPPC:DSPE-PEG (96:4) and DPPC:MPPC:DSPE-PEG (86:10:4) LUVETs, Figure S3. Key procedural steps in animal surgery and implantation of the optical chamber, Figure S4. Optical properties of the emission light source and fluorophores for intravital fluorescence microscopy, Figure S5. Custom-built laser system used to induce thrombosis in hamster dorsal venules during intravital fluorescence microscopy, Figure S6. Saturation and lightness settings per hue used for the analysis of green fluorescence in laser-induced thrombi in Photoshop, Figure S7. Differential scanning calorimetry thermograms of TA-encapsulating LUVETs of different phospholipid compositions, Figure S8. Calcein leakage at 4 °C from DPPC:DSPE-PEG (96:4) and DPPC:MPPC:DSPE-PEG (86:10:4) LUVETs containing calcein at iso-osmolar and hypo-osmolar conditions relative to physiological buffer and plasma, Figure S9. Calcein leakage at body temperature (37 °C) from DPPC:DSPE-PEG (96:4) and DPPC:MPPC:DSPE-PEG (86:10:4) LUVETs loaded with a hypo-osmolar calcein solution relative to physiological buffer and plasma, Figure S10. Calcein leakage during a 20 °C → 47.0 °C temperature ramp from DPPC:DSPE-PEG (96:4) and DPPC:MPPC:DSPE-PEG (86:10:4) LUVETs loaded with a hypo-osmolar calcein solution, Figure S11. Representative flow cytograms of resting and convulxin-activated hamster and human platelets incubated with 5(6)-carboxyfluorescein, calcein, and anti-CD62P-FITC, Figure S12. Thrombus kinetics in the negative control group following multiple laser pulses, Figure S13. Time lapse series of a laser-induced thrombus intravitally stained with FITC-conjugated anti-CD62P antibodies, Figure S14. Fluorescence of FITC-conjugated anti-CD62P antibodies in various tissues, Figure S15. Fluorescence of 5(6)-carboxyfluorescein in various tissues, Figure S16. Fluorescence of CF-encapsulating DSPC:DSPE-PEG (96:4) LUVETs in various tissues, Figure S17. Fluorescence of DPPC:NBD-PC:DSPE-PEG (91:5:4) LUVETs in various tissues.

## References

[1] Tan W., Wang J., Zhou F., Gao L., Yin R., Liu H., Sukanthanag A., Wang G., Mihm M.C., Chen D.B. (2017). Coexistence of Eph receptor B1 and ephrin B2 in port-wine stain endothelial progenitor cells contributes to clinicopathological vasculature dilatation. Br. J. Dermatol..

[2] Nguyen V., Hochman M., Mihm M.C., Nelson J.S., Tan W. (2019). The Pathogenesis of Port Wine Stain and Sturge Weber Syndrome: Complex Interactions between Genetic Alterations and Aberrant MAPK and PI3K Activation. Int. J. Mol. Sci..

[3] Fiskerstrand E.J., Svaasand L.O., Kopstad G., Ryggen K., Aase S. (1996). Photothermally induced vessel-wall necrosis after pulsed dye laser treatment: Lack of response in port-wine stains with small sized or deeply located vessels. J. Investig. Dermatol..

[4] Anderson R.R., Parrish J.A. (1983). Selective photothermolysis: Precise microsurgery by selective absorption of pulsed radiation. Science.

[5] Heger M., Beek J., Stenback K., Faber D., van Gemert M., Ince C. (2005). Darkfield orthogonal polarized spectral imaging for studying endovascular laser-tissue interactions in vivo-a preliminary study. Opt. Express.

[6] Bezemer R., Heger M., van den Wijngaard J.P., Mordon S.R., van Gemert M.J., Beek J.F. (2007). Laser-induced (endo)vascular photothermal effects studied by combined brightfield and fluorescence microscopy in hamster dorsal skin fold venules. Opt. Express.

[7] Heger M., Beek J.F., Moldovan N.I., van der Horst C.M., van Gemert M.J. (2005). Towards optimization of selective photothermolysis: Prothrombotic pharmaceutical agents as potential adjuvants in laser treatment of port wine stains. A theoretical study. Thromb. Haemost..

[8] Hohenleutner U., Hilbert M., Wlotzke U., Landthaler M. (1995). Epidermal damage and limited coagulation depth with the flashlamp-pumped pulsed dye laser: A histochemical study. J. Investig. Dermatol..

[9] Chen J.K., Ghasri P., Aguilar G., van Drooge A.M., Wolkerstorfer A., Kelly K.M., Heger M. (2012). An overview of clinical and experimental treatment modalities for port wine stains. J. Am. Acad. Dermatol..

[10] Van Raath M.I., Chohan S., Wolkerstorfer A., van der Horst C., Storm G., Heger M. (2019). Port wine stain treatment outcomes have not improved over the past three decades. J. Eur. Acad. Dermatol. Venereol..

[11] Heger M., Bezemer R., Huertas-Perez J.F., Dekker H., Beek J.F. (2010). Endovascular laser-tissue interactions redefined: Shining light on novel windows of therapeutic opportunity beyond selective photothermolysis. Photomed. Laser Surg..

[12] Van Raath M.I., Bambach C.A., Dijksman L.M., Wolkerstorfer A., Heger M. (2018). Prospective analysis of the port-wine stain patient population in the Netherlands in light of novel treatment modalities. J. Cosmet. Laser Ther..

[13] Aguilar G., Choi B., Broekgaarden M., Yang O., Yang B., Ghasri P., Chen J.K., Bezemer R., Nelson J.S., van Drooge A.M. (2012). An overview of three promising mechanical, optical, and biochemical engineering approaches to improve selective photothermolysis of refractory port wine stains. Ann. Biomed. Eng..

[14] Van Raath M.I., van Amesfoort J.E., Hermann M., Ince Y., Zwart M.J., Echague A.V., Chen Y., Ding B., Huang X., Storm G. (2019). Site-specific pharmaco-laser therapy: A novel treatment modality for refractory port wine stains. J. Clin. Transl. Res..

[15] Heger M., Salles I.I., Bezemer R., Cloos M.A., Mordon S.R., Begu S., Deckmyn H., Beek J.F. (2011). Laser-induced primary and secondary hemostasis dynamics and mechanisms in relation to selective photothermolysis of port wine stains. J. Dermatol. Sci..

[16] Van Raath M.I., Weijer R., Nguyen G.H., Choi B., de Kroon A.I., Heger M. (2016). Tranexamic Acid-Encapsulating Thermosensitive Liposomes for Site-Specific Pharmaco-Laser Therapy of Port Wine Stains. J. Biomed. Nanotechnol..

[17] Huertas-Perez J.F., Heger M., Dekker H., Krabbe H., Lankelma J., Ariese F. (2007). Simple, rapid, and sensitive liquid chromatography-fluorescence method for the quantification of tranexamic acid in blood. J. Chromatogr. A.

[18] Houston B.L., Uminski K., Mutter T., Rimmer E., Houston D.S., Menard C.E., Garland A., Ariano R., Tinmouth A., Abou-Setta A.M. (2020). Efficacy and Safety of Tranexamic Acid in Major Non-Cardiac Surgeries at High Risk for Transfusion: A Systematic Review and Meta-Analysis. Transfus. Med. Rev..

[19] Fusca L., Perelman I., Fergusson D., Boutet M., Chen I. (2019). The Effectiveness of Tranexamic Acid at Reducing Blood Loss and Transfusion Requirement for Women Undergoing Myomectomy: A Systematic Review and Meta-analysis. J. Obstet. Gynaecol. Can..

[20] Reed M.R., Woolley L.T. (2014). Uses of tranexamic acid. Contin. Educ. Anaesth. Crit. Care Pain.

[21] Smithies D.J., van Gemert M.J., Hansen M.K., Milner T.E., Nelson J.S. (1997). Three-dimensional reconstruction of port wine stain vascular anatomy from serial histological sections. Phys. Med. Biol..

[22] Selim M.M., Kelly K.M., Nelson J.S., Wendelschafer-Crabb G., Kennedy W.R., Zelickson B.D. (2004). Confocal microscopy study of nerves and blood vessels in untreated and treated port wine stains: Preliminary observations. Dermatol. Surg..

[23] Heger M., Salles I.I., van Vuure W., Hamelers I.H., de Kroon A.I., Deckmyn H., Beek J.F. (2009). On the interaction of fluorophore-encapsulating PEGylated lecithin liposomes with hamster and human platelets. Microvasc. Res..

[24] Heger M., Salles I.I., de Kroon A.I., Deckmyn H. (2009). Platelets and PEGylated lecithin liposomes: When stealth is allegedly picked up on the radar (and eaten). Microvasc. Res..

[25] Rouser G., Fkeischer S., Yamamoto A. (1970). Two dimensional then layer chromatographic separation of polar lipids and determination of phospholipids by phosphorus analysis of spots. Lipids.

[26] Udenfriend S., Stein S., Bohlen P., Dairman W., Leimgruber W., Weigele M. (1972). Fluorescamine: A reagent for assay of amino acids, peptides, proteins, and primary amines in the picomole range. Science.

[27] Broekgaarden M., de Kroon A.I., Gulik T.M., Heger M. (2014). Development and in vitro proof-of-concept of interstitially targeted zinc- phthalocyanine liposomes for photodynamic therapy. Curr. Med. Chem..

[28] Danaei M., Dehghankhold M., Ataei S., Hasanzadeh Davarani F., Javanmard R., Dokhani A., Khorasani S., Mozafari M.R. (2018). Impact of Particle Size and Polydispersity Index on the Clinical Applications of Lipidic Nanocarrier Systems. Pharmaceutics.

[29] Zuidam N.J.d.V.R., Crommelin D.J., Torchilin V.P., Weissig V. (2003). Characterization of liposomes. Liposomes.

[30] Nagle J.F., Tristram-Nagle S. (2000). Lipid bilayer structure. Curr. Opin. Struct. Biol..

[31] Majewski J., Kuhl T.L., Kjaer K., Gerstenberg M.C., Als-Nielsen J., Israelachvili J.N., Smith G.S. (1998). X-ray Synchrotron Study of Packing and Protrusions of Polymer−Lipid Monolayers at the Air−Water Interface. J. Am. Chem. Soc..

[32] Chi L.M., Wu W.G. (1990). Effective bilayer expansion and erythrocyte shape change induced by monopalmitoyl phosphatidylcholine. Quantitative light microscopy and nuclear magnetic resonance spectroscopy measurements. Biophys J..

[33] Faller R., Marrink S.J. (2004). Simulation of domain formation in DLPC-DSPC mixed bilayers. Langmuir.

[34] Wheeler J.J., Palmer L., Ossanlou M., MacLachlan I., Graham R.W., Zhang Y.P., Hope M.J., Scherrer P., Cullis P.R. (1999). Stabilized plasmid-lipid particles: Construction and characterization. Gene Ther..

[35] De Graaf W., Heger M., Spruijt O., Maas A., de Bruin K., Hoekstra R., Bennink R.J., van Gulik T.M. (2012). Quantitative assessment of liver function after ischemia-reperfusion injury and partial hepatectomy in rats. J. Surg. Res..

[36] Rowan A.N., Salem D.J. (2001). The State of the Animals.

[37] Stenberg P.E., McEver R.P., Shuman M.A., Jacques Y.V., Bainton D.F. (1985). A platelet alpha-granule membrane protein (GMP-140) is expressed on the plasma membrane after activation. J. Cell Biol..

[38] Heger M., Salles I.I., van Vuure W., Deckmyn H., Beek J.F. (2009). Fluorescent labeling of platelets with polyanionic fluorescein derivatives. Anal. Quant. Cytol. Histol..

[39] Reiniers M.J., van Golen R.F., Bonnet S., Broekgaarden M., van Gulik T.M., Egmond M.R., Heger M. (2017). Preparation and Practical Applications of 2′,7′-Dichlorodihydrofluorescein in Redox Assays. Anal. Chem..

[40] Guerci P., Ergin B., Uz Z., Ince Y., Westphal M., Heger M., Ince C. (2019). Glycocalyx Degradation Is Independent of Vascular Barrier Permeability Increase in Nontraumatic Hemorrhagic Shock in Rats. Anesth. Analg..

[41] Dos Santos N., Allen C., Doppen A.M., Anantha M., Cox K.A., Gallagher R.C., Karlsson G., Edwards K., Kenner G., Samuels L. (2007). Influence of poly(ethylene glycol) grafting density and polymer length on liposomes: Relating plasma circulation lifetimes to protein binding. Biochim. Biophys. Acta.

[42] Mastrotto F., Brazzale C., Bellato F., De Martin S., Grange G., Mahmoudzadeh M., Magarkar A., Bunker A., Salmaso S., Caliceti P. (2020). In Vitro and in Vivo Behavior of Liposomes Decorated with PEGs with Different Chemical Features. Mol. Pharm..

[43] Maruyama K., Yuda T., Okamoto A., Kojima S., Suginaka A., Iwatsuru M. (1992). Prolonged circulation time in vivo of large unilamellar liposomes composed of distearoyl phosphatidylcholine and cholesterol containing amphipathic poly(ethylene glycol). Biochim. Biophys. Acta.

[44] Watabe N., Ishida Y., Ochiai A., Tokuoka Y., Kawashima N. (2007). Oxidation decomposition of unsaturated fatty acids by singlet oxygen in phospholipid bilayer membranes. J. Oleo Sci..

[45] Mori A., Klibanov A.L., Torchilin V.P., Huang L. (1991). Influence of the steric barrier activity of amphipathic poly(ethyleneglycol) and ganglioside GM1 on the circulation time of liposomes and on the target binding of immunoliposomes in vivo. FEBS Lett..

[46] Manni M.M., Tiberti M.L., Pagnotta S., Barelli H., Gautier R., Antonny B. (2018). Acyl chain asymmetry and polyunsaturation of brain phospholipids facilitate membrane vesiculation without leakage. Elife.

[47] Gaber M.H., Wu N.Z., Hong K., Huang S.K., Dewhirst M.W., Papahadjopoulos D. (1996). Thermosensitive liposomes: Extravasation and release of contents in tumor microvascular networks. Int. J. Radiat Oncol. Biol. Phys..

[48] Han S.-M., Na Y.-G., Lee H.-S., Son G.-H., Jeon S.-H., Bang K.-H., Kim S.-J., Lee H.-J., Cho C.-W. (2018). Improvement of cellular uptake of hydrophilic molecule, calcein, formulated by liposome. J. Pharm. Investig..

[49] Mulik R., Kulkarni V., Murthy R.S. (2009). Chitosan-based thermosensitive hydrogel containing liposomes for sustained delivery of cytarabine. Drug Dev. Ind. Pharm..

[50] Dicko A., Kwak S., Frazier A.A., Mayer L.D., Liboiron B.D. (2010). Biophysical characterization of a liposomal formulation of cytarabine and daunorubicin. Int. J. Pharm..

[51] Lim S.K., Shin D.H., Choi M.H., Kim J.S. (2014). Enhanced antitumor efficacy of gemcitabine-loaded temperature-sensitive liposome by hyperthermia in tumor-bearing mice. Drug Dev. Ind. Pharm..

[52] Tong Q., Li H., Li W., Chen H., Shu X., Lu X., Wang G. (2011). In vitro and in vivo anti-tumor effects of gemcitabine loaded with a new drug delivery system. J. Nanosci. Nanotechnol..

[53] Liu J.J., Hong R.L., Cheng W.F., Hong K., Chang F.H., Tseng Y.L. (2002). Simple and efficient liposomal encapsulation of topotecan by ammonium sulfate gradient: Stability, pharmacokinetic and therapeutic evaluation. Anticancer Drugs.

[54] Dadashzadeh S., Vali A.M., Rezaie M. (2008). The effect of PEG coating on in vitro cytotoxicity and in vivo disposition of topotecan loaded liposomes in rats. Int. J. Pharm..

[55] Lim C.B., Abuzar S.M., Karn P.R., Cho W., Park H.J., Cho C.W., Hwang S.J. (2019). Preparation, Characterization, and In Vivo Pharmacokinetic Study of the Supercritical Fluid-Processed Liposomal Amphotericin B. Pharmaceutics.

[56] Moribe K., Tanaka E., Maruyama K., Iwatsuru M. (1998). Enhanced encapsulation of amphotericin B into liposomes by complex formation with polyethylene glycol derivatives. Pharm. Res..

[57] Javed I., Hussain S.Z., Ullah I., Khan I., Ateeq M., Shahnaz G., Rehman H.U., Razi M.T., Shah M.R., Hussain I. (2015). Synthesis, characterization and evaluation of lecithin-based nanocarriers for the enhanced pharmacological and oral pharmacokinetic profile of amphotericin B. J. Mater. Chem. B.

[58] Li L., ten Hagen T.L., Hossann M., Suss R., van Rhoon G.C., Eggermont A.M., Haemmerich D., Koning G.A. (2013). Mild hyperthermia triggered doxorubicin release from optimized stealth thermosensitive liposomes improves intratumoral drug delivery and efficacy. J. Control. Release.

[59] Maruyama K., Unezaki S., Takahashi N., Iwatsuru M. (1993). Enhanced delivery of doxorubicin to tumor by long-circulating thermosensitive liposomes and local hyperthermia. Biochim. Biophys. Acta.

[60] Tagami T., Ernsting M.J., Li S.D. (2011). Efficient tumor regression by a single and low dose treatment with a novel and enhanced formulation of thermosensitive liposomal doxorubicin. J. Control. Release.

[61] Shen S., Huang D., Cao J., Chen Y., Zhang X., Guo S., Ma W., Qi X., Ge Y., Wu L. (2019). Magnetic liposomes for light-sensitive drug delivery and combined photothermal-chemotherapy of tumors. J. Mater. Chem. B.

[62] Han B., Yang Y., Chen J., Tang H., Sun Y., Zhang Z., Wang Z., Li Y., Li Y., Luan X. (2020). Preparation, Characterization, and Pharmacokinetic Study of a Novel Long-Acting Targeted Paclitaxel Liposome with Antitumor Activity. Int. J. Nanomed..

[63] Yang T., Cui F.D., Choi M.K., Cho J.W., Chung S.J., Shim C.K., Kim D.D. (2007). Enhanced solubility and stability of PEGylated liposomal paclitaxel: In vitro and in vivo evaluation. Int. J. Pharm..

[64] Mayer L.D., Bally M.B., Loughrey H., Masin D., Cullis P.R. (1990). Liposomal vincristine preparations which exhibit decreased drug toxicity and increased activity against murine L1210 and P388 tumors. Cancer Res..

[65] Tartau L., Cazacu A., Melnig V. (2012). Ketoprofen-liposomes formulation for clinical therapy. J. Mater. Sci. Mater. Med..

[66] Maestrelli F., Gonzalez-Rodriguez M.L., Rabasco A.M., Mura P. (2006). Effect of preparation technique on the properties of liposomes encapsulating ketoprofen-cyclodextrin complexes aimed for transdermal delivery. Int. J. Pharm..

[67] Samanta K., Setua S., Kumari S., Jaggi M., Yallapu M.M., Chauhan S.C. (2019). Gemcitabine Combination Nano Therapies for Pancreatic Cancer. Pharmaceutics.

[68] Hossann M., Syunyaeva Z., Schmidt R., Zengerle A., Eibl H., Issels R.D., Lindner L.H. (2012). Proteins and cholesterol lipid vesicles are mediators of drug release from thermosensitive liposomes. J. Control. Release.

[69] Marsh D., Watts A., Knowles P.F. (1977). Cooperativity of the phase transition in single- and multibilayer lipid vesicles. Biochim. Biophys. Acta.

[70] Nagle J.F. (1980). Theory of the main lipid bilayer phase transition. Ann. Rev. Phys. Chem..

[71] Marsh D. (1991). General features of phospholipid phase transitions. Chem. Phys. Lipids.

[72] Nagle J.F., Scott H.L. (1978). Lateral compressibility of lipid mono- and bilayers. Theory of membrane permeability. Biochim. Biophys. Acta.

[73] Ruppel D.S.E. (1983). On defects in different phases of two-dimensional lipid bilayers. J. Phys..

[74] Hosokawa T., Sami M., Kato Y., Hayakawa E. (2003). Alteration in the temperature-dependent content release property of thermosensitive liposomes in plasma. Chem. Pharm. Bull. (Tokyo).

[75] Panagi Z.A.K., Evangelatos G., Ithakissios D.S. (1998). Protein-induced CF release from liposomes in vitro and its correlation with the BLOOD/RES biodistribution of liposomes. Int. J. Pharm..

[76] Burke C., Dreher M.R., Negussie A.H., Mikhail A.S., Yarmolenko P., Patel A., Skilskyj B., Wood B.J., Haemmerich D. (2018). Drug release kinetics of temperature sensitive liposomes measured at high-temporal resolution with a millifluidic device. Int. J. Hyperth..

[77] Kneidl B., Peller M., Winter G., Lindner L.H., Hossann M. (2014). Thermosensitive liposomal drug delivery systems: State of the art review. Int. J. Nanomed..

[78] Picetti R., Shakur-Still H., Medcalf R.L., Standing J.F., Roberts I. (2019). What concentration of tranexamic acid is needed to inhibit fibrinolysis? A systematic review of pharmacodynamics studies. Blood Coagul. Fibrinolysis.

[79] Weijer R., Broekgaarden M., Kos M., van Vught R., Rauws E.A., Breukink E.J., van Gulik T.M., Storm G., Heger M. (2015). Enhancing photodynamic therapy of refractory solid cancers: Combining second-generation photosensitizers with multi-targeted liposomal delivery. J. Photochem. Photobiol. C.

[80] Weijer R., Broekgaarden M., van Golen R.F., Bulle E., Nieuwenhuis E., Jongejan A., Moerland P.D., van Kampen A.H., van Gulik T.M., Heger M. (2015). Low-power photodynamic therapy induces survival signaling in perihilar cholangiocarcinoma cells. BMC Cancer.

[81] Weijer R., Clavier S., Zaal E.A., Pijls M.M., van Kooten R.T., Vermaas K., Leen R., Jongejan A., Moerland P.D., van Kampen A.H. (2017). Multi-OMIC profiling of survival and metabolic signaling networks in cells subjected to photodynamic therapy. Cell Mol. Life Sci..

[82] Broekgaarden M., van Vught R., Oliveira S., Roovers R.C., van Bergen en Henegouwen P.M., Pieters R.J., Van Gulik T.M., Breukink E., Heger M. (2016). Site-specific conjugation of single domain antibodies to liposomes enhances photosensitizer uptake and photodynamic therapy efficacy. Nanoscale.

[83] Broekgaarden M., Weijer R., Krekorian M., van den Ijssel B., Kos M., Alles L.K., van Wijk A.C., Bikadi Z., Hazai E., van Gulik T.M. (2016). Inhibition of hypoxia-inducible factor 1 with acriflavine sensitizes hypoxic tumor cells to photodynamic therapy with zinc phthalocyanine-encapsulating cationic liposomes. Nano Res..

[84] Frimer A.A. (1979). The reaction of singlet oxygen with olefins: The question of mechanism. Chem. Rev..

[85] Ryter S.W., Tyrrell R.M. (1998). Singlet molecular oxygen ((1)O2): A possible effector of eukaryotic gene expression. Free Radic. Biol. Med..

[86] Davies M.J. (2004). Reactive species formed on proteins exposed to singlet oxygen. Photochem. Photobiol. Sci..

[87] Campbell P.I. (1983). Toxicity of some charged lipids used in liposome preparations. Cytobios.

[88] Mayhew E., Ito M., Lazo R. (1987). Toxicity of non-drug-containing liposomes for cultured human cells. Exp. Cell Res..

[89] Sevanian A.H.P. (1985). Mechanisms and consequences of lipid peroxidation in biological systems. Ann. Rev. Nutr..

[90] Lakmaker O., Pickering J.W., van Gemert M.J. (1993). Modeling the color perception of port wine stains and its relation to the depth of laser coagulated blood vessels. Lasers Surg. Med..

[91] Verkruysse W., Lucassen G.W., van Gemert M.J. (1999). Simulation of color of port wine stain skin and its dependence on skin variables. Lasers Surg. Med..

[92] Choi B., Tan W., Jia W., White S.M., Moy W.J., Yang B.Y., Zhu J., Chen Z., Kelly K.M., Nelson J.S. (2016). The Role of Laser Speckle Imaging in Port-Wine Stain Research: Recent Advances and Opportunities. IEEE J. Sel. Top. Quantum. Electron..

[93] Yang B., Yang O., Guzman J., Nguyen P., Crouzet C., Osann K.E., Kelly K.M., Nelson J.S., Choi B. (2015). Intraoperative, real-time monitoring of blood flow dynamics associated with laser surgery of port wine stain birthmarks. Lasers Surg. Med..

[94] Weissmann G., Brand A., Franklin E.C. (1974). Interaction of immunoglobulins with liposomes. J. Clin. Investig..

[95] Mittag J.J., Kneidl B., Preibeta T., Hossann M., Winter G., Wuttke S., Engelke H., Radler J.O. (2017). Impact of plasma protein binding on cargo release by thermosensitive liposomes probed by fluorescence correlation spectroscopy. Eur. J. Pharm. Biopharm..

[96] Wohner N. (2008). Role of cellular elements in thrombus formation and dissolution. Cardiovasc. Hematol. Agents Med. Chem..

