# Supplementary material for: In Vivo Assessment of Thermosensitive Liposomes for the Treatment of Port Wine Stains by Antifibrinolytic Site-Specific Pharmaco-Laser Therapy
Source: Pharmaceutics. 2020 Jun 25;12(6):591. doi: 10.3390/pharmaceutics12060591 (PMC7356038; doi:10.3390/pharmaceutics12060591)
Supplement: Supplementary file 1 [file pharmaceutics-12-00591-s001.pdf]

pharmaceutics

Supplementary Materials: In Vivo Assessment of Thermosensitive Liposomes for the Treatment of Port Wine Stains by Antifibrinolytic Site-Specific Pharmaco-Laser Therapy

Mingjuan Li, M. Ingmar van Raath, Shervin Khakpour, Ahmet Seçilir, Bart C. Sliggers, Xuan Huang, Baoyue Ding, Gert Storm, René R. van der Hulst, Anton I.P.M. de Kroon and Michal Heger

S1. List of abbreviations

A	area of the membrane occupied by one lipid
amu	atomic mass units
$A_{weighed}$	weighed area
CD62P	P-selectin
CF	5(6)-carboxyfluorescein
$CHCl_3$	chloroform
C_{TA}	endovesicular TA concentration
DOPE-PEG2000	1,2-dioleoyl-sn-glycero-3-phosphoethanolamine-N-[methoxy(polyethylene glycol)-2000]
DOPE-PEG5000	1,2-dioleoyl-sn-glycero-3-phosphoethanolamine-N-[methoxy(polyethylene glycol)-5000]
DPPC	1,2-dipalmitoyl-sn-glycero-3-phosphocholine
DSPC	1,2-distearoyl-sn-glycero-3-phosphocholine
DSPE-PEG2000	L-α-phosphatidylethanolamine, distearoyl methoxypolyethylene glycol conjugate
E_{eff}	encapsulation efficiency
eV_t	trapped volume per vesicle
F	formulation
F_{ave}	mean fluorescence intensity over the indicated time interval
fc	final concentration
FITC	fluorescein isothiocyanate
F_t	fluorescence intensity at time point t
GMP	good manufacturing practice
HCl	hydrochloric acid
HEPES	4-(2-hydroxyethyl)piperazine-1-ethanesulfonic acid
IgG	immunoglobulin G
Kcps	kilocounts per second
l_{im}	cumulative number of lipids in the inner membrane leaflet
l_{om}	cumulative number of lipids in the outer membrane leaflet
LUVET	large unilamellar vesicles prepared by extrusion technique
MPPC	1-palmitoyl-2-hydroxy-sn-glycero-3-phosphocholine (lysoPC)
N	Avogadro constant
NaCl	sodium chloride
NaOH	sodium hydroxide
NBD-PC	1-palmitoyl-2-{12-[(7-nitro-2-1,3-benzoxadiazol-4-yl)amino]dodecanoyl}-sn-glycero-3-phosphocholine
N.S.	not specified
PBS	phosphate buffered saline
PEG	polyethylene glycol
PPP	platelet-poor plasma
PRP	platelet-rich plasma
PWS	port wine stain

Q_{TA}	quantity of TA molecules per vesicle
r	radius
R	release rate
RT	room temperature
r_v	vesicle radius
SDPC	1-palmitoyl-2-docosahexaenoyl-sn-glycero-3-phosphocholine
SSPLT	site-specific pharmaco-laser therapy
T	temperature
TA	tranexamic acid
T_m	phase transition temperature
TX100	Triton X-100
V_t	trapped volume (L·mole^{-1} lipid)

S2. Materials and methods

S2.1. Materials

Table S1. List of chemicals and reagents.

Compound	Purity	Supplier	Additional information
**Phospholipids and Surfactants**			
DPPC	>99%	Avanti Polar Lipids$	Product # 850355P, 16:0 PC, MW = 734.039 g/mol; dissolved in $CHCl_3$ at ~20 mM stock concentration, stored under N_2 atmosphere at −20 °C
DSPC	>99%	Avanti Polar Lipids$	Product # 810131P, 18:0 PC, MW = 790.145 g/mol; dissolved in $CHCl_3$ at ~13 mM stock concentration, stored under N_2 atmosphere at −20 °C
MPPC	>99%	Avanti Polar Lipids$	Product # 855675P, 16:0 lysoPC, MW = 495.630 g/mol; dissolved in $CHCl_3$ at ~20 mM stock concentration, stored under N_2 atmosphere at −20 °C
SDPC	>99%	Avanti Polar Lipids$	Product # 850461C, 16:0-22:6 PC, MW = 834.156 g/mol; dissolved in $CHCl_3$ at 12 mM stock concentration (by supplier), stored under N_2 atmosphere at −20 °C
DSPE-PEG2000	>98%	Sigma-Aldrich#	Product # P7840, 18:0 PC-PEG, MW = 2805.5 g/mol; average PEG molecular mass of 2,000 amu, dissolved in $CHCl_3$ at ~4 mM stock concentration, stored under N_2 atmosphere at −20 °C
DOPE-PEG2000	>99%	Avanti Polar Lipids$	Product # 880130P, 18:1 PE-PEG, MW = 2799.760 g/mol; average PEG molecular mass of 5,000 amu, dissolved in $CHCl_3$ at ~4 mM stock concentration, stored under N_2 atmosphere at −20 °C
DOPE-PEG5000	>99%	Avanti Polar Lipids$	Product # 880230P, 18:1 PE-PEG, MW = 5797.100 g/mol; average PEG molecular mass of 5,000 amu, dissolved in $CHCl_3$ at ~4 mM stock concentration, stored under N_2 atmosphere at −20 °C
NBD-PC	>99%	Avanti Polar Lipids$	Product # 810131P, 16:0-12:0 NBD PC, MW = 856.038 g/mol; dissolved in $CHCl_3$ at 10 mM stock concentration, stored under N_2 atmosphere at −20 °C
TX-100	BioXtra	Sigma-Aldrich#	Product # T9284, average MW = 625 g/mol; dissolved in MilliQ at 5% (v/v) stock concentration, stored at room temperature
**Liposomal Encapsulants, Buffers, and Osmolarity Regulators**			
Calcein	N.S.	Sigma-Aldrich#	Product # C0875, average MW = 622.53 g/mol; stored at room temperature in the dark following dissolution as described in the main text
HEPES (sodium salt)	≥99.5%	Sigma-Aldrich#	Product # H7006, MW = 260.29 g/mol; dissolved at 10 mM concentration in MilliQ containing 0.88% (w/v) NaCl, pH adjusted to 7.4 with HCl to prepare physiological buffer
NaCl	99.99%	Merck KGaA*	Product # 106406, MW = 58.44 g/mol; added to 10 mM HEPES solution (to adjust osmolarity to physiological level) to prepare physiological buffer
TA	~98%	Fluka†	Product # 08455, MW = 157.21 g/mol; dissolved at 318 mM concentration in MilliQ containing 10 mM HEPES, adjusted to pH = 7.4 with NaOH, stored under N_2 atmosphere in the dark at room temperature
**Reagents**			
Ammonium heptamolybdate tetrahydrate	≥99.0%	Merck KGaA*	Product # 101182, MW = 1235.86 g/mol; dissolved in MilliQ at 1.25% (w/v) concentration and stored at room temperature

Ascorbic acid	≥99%	Acros Organics‡	Product # 105025000, MW = 176.13 g/mol; dissolved in MilliQ at 5% (w/v) concentration and stored under N_2 atmosphere in the dark at 4 °C
Fluorescamine (4-phenylspiro-[furan-2(3H),1-phthalan]-3,3'-dione)	≥98%	Sigma-Aldrich#	Product # F9015, MW = 278.26 g/mol; dissolved in acetone at 1.08 mM stock concentration, stored under N_2 atmosphere in the dark at 4 °C
Perchloric acid	70.0–72.0%	Sigma-Aldrich#	Product # 30755
**Solvents**			
Acetone	≥99.5%	Sigma-Aldrich#	Product # 179124
$CHCl_3$	≥99%	Sigma-Aldrich#	Product # 288306
**pH Regulators**			
HCl	ACS reagent	Sigma-Aldrich#	Product # 258148
NaOH	≥99.0%	Merck KGaA*	Product # 106498, dissolved in MilliQ at 1 M concentration
**Flow Cytometry and In Vivo Experiments**			
PBS	N.S.	Fresenius Kabi§	Sterile, for infusion
Sodium citrate	≥99.0%	Sigma-Aldrich#	Product # 71498, purum p. a., anhydrous, MW = 214.11 g/mol; dissolved in MilliQ at 3.8% stock concentration and stored at 4 °C in the dark
Convulxin	N.S.	Kordia Life Sciencesɫ	Dissolved in MilliQ water at a 10 µg/mL concentration, aliquoted (50 µL), and stored at -80 °C.
CF	≥95.0%	Sigma-Aldrich#	Product # 21877, MW = 376.32 g/mol; stored at room temperature in the dark following dissolution as described in the main text
Anti-CD62P antibodies, FITC-conjugated	0.5 mg/mL	Research Diagnostics+	Rat anti-mouse, clone RB40.34, cross-reactivity with hamster P-selectin (CD62P) confirmed in [1], stored at 4 °C in the dark
Anti-$IgG_{1\kappa}$ antibodies, FITC-conjugated	0.5 mg/mL	Research Diagnostics+	Rat anti-mouse, clone R3-34, isotype control antibodies for rat anti-mouse CD62P antibodies, stored at 4 °C in the dark

$ Alabaster, AL, USA, # St. Louis, MO, USA, † Buchs, Switzerland, * Darmstadt, Germany, ‡ Geel, Belgium, ɫ Leiden, The Netherlands, § Bad Homburg, Germany, + Flanders, NJ, USA.

Systemically administered liposomes encapsulating compounds in the aqueous phase should have an aqueous compartment that is iso-osmolar with respect to blood (0.292 osmol·kg^{-1}) to prevent membrane destabilization due to large osmotic gradients [2] and consequent leakage of encapsulants. To that end, osmolarities were determined for 0-154 mM NaCl in MilliQ (pH = 7.4, adjusted with 0.01 M NaOH in MilliQ), 0-684 mM NaCl in 10 mM HEPES buffer (pH = 7.4, adjusted with 10 M NaOH in MilliQ), and 0-318 mM TA in 10 mM HEPES buffer (pH = 7.4, adjusted with 10 M NaOH in MilliQ) using a cryoscopic osmometer (Osmomat 030, Gonotec, Berlin, Germany).

For the NaCl in MilliQ group, MilliQ water was adjusted to pH = 7.4 with 0.01 M NaOH, yielding an osmolarity of 0.0061 osmol/kg as a result of the pH adjustment. A starting solution of 0.9% (w/v) NaCl (154.0 mM) was prepared with the pH-adjusted MilliQ water. The starting solution was diluted to 115.5 mM, 77 mM, 38.5 mM, and 19.25 mM NaCl with the pH-adjusted MilliQ water.

For the NaCl in 10 mM HEPES buffer group, HEPES was dissolved in MilliQ water at a 10-mM concentration and the solution was adjusted to pH = 7.4 with 10 M NaOH in MilliQ, accounting for an osmolarity of 0.0244 osmol/kg. Next, a 5% (w/v) NaCl starting solution (855.5 mM) was prepared in the pH-adjusted HEPES buffer. The pH-adjusted HEPES buffer was used to dilute the 5% NaCl starting solution to 4% NaCl (684.4 mM), 3% NaCl (513.3 mM), 2% NaCl (342.2 mM), and 1% NaCl (171.1 mM) solutions. All listed concentrations were measured, but the osmolarity is only reported for the 0-4% NaCl concentration range.

For the TA in 10 mM HEPES group, HEPES was dissolved in MilliQ water at a 10-mM concentration and the solution was adjusted to pH = 7.4 with 10 M NaOH in MilliQ, producing an osmolarity of 0.0214 osmol/kg. Next, a 10% (w/v) TA (636.1 mM) starting solution was prepared in

the pH-adjusted HEPES buffer. The pH-adjusted HEPES buffer was used to dilute the 10% TA starting solution to 9% TA (572.5 mM), 8% TA (508.9 mM), 7% TA (445.3 mM), 6% TA (381.7 mM), 5% TA (318.1 mM), 4% TA (254.5 mM), 3% TA (190.8 mM), 2% TA (127.2 mM), and 1% TA (63.6 mM) concentrations. All listed concentrations were measured, but the osmolarity is only reported for the 0-5% TA concentration range.

For the calcein in MilliQ group, a 50-mM calcein solution was prepared by adding 350 mg of calcein to 10 mL of MilliQ water containing 50 mM NaOH. The pH was raised further by drop-wise addition of 10 M NaOH in MilliQ during continuous stirring with a magnetic stirrer and occasional heating until the calcein was completely dissolved. Next, the solution was allowed to cool down and thereafter gradually acidified by dropwise addition of 3.7% HCl until pH = 7.4 was reached during continuous stirring and real-time pH measurement. The final solution was serially diluted to a concentration of 0.8 mM with MilliQ that had been adjusted to pH = 7.4 with 0.01 M NaOH (yielding 0.0059 osmol/kg). It should be noted that the 50-mM calcein solution is an approximate concentration based on the additional volume that was added to the solution for solubilization and pH adjustments. For these experiments it was more important to achieve a self-quenching calcein solution with known osmolarity that could be adjusted to iso-osmolar levels using e.g., NaCl in pH-neutral MilliQ water.

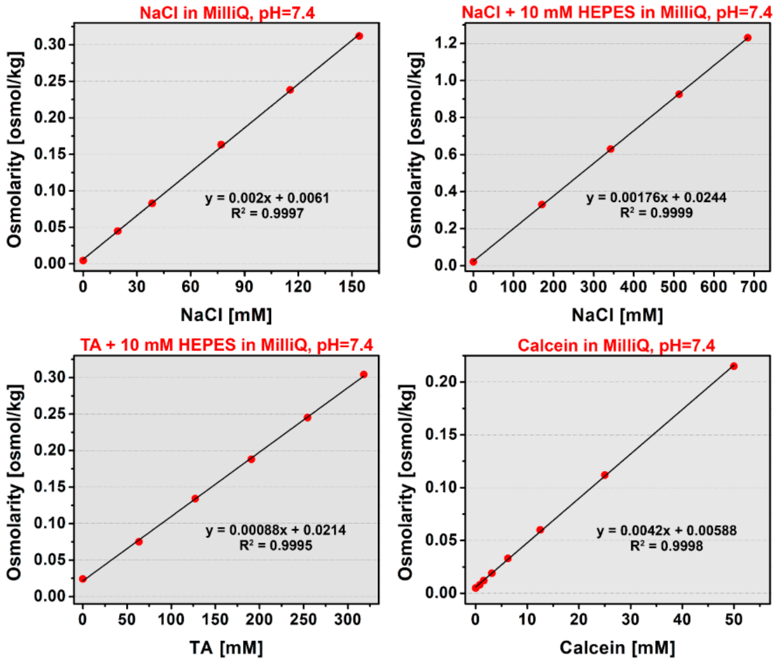

Figure S1. Osmolarities of the different solutions used for liposome and buffer preparation, plotted as a function of solute concentration. Data were fitted with a linear function. The equation is provided in the plots along with the goodness of fit (R^2) values.

The osmolarity of different concentrations calcein and NaCl dissolved in pH-adjusted MilliQ (pH = 7.4) was also measured (Figure S1) for heat-induced release assays in aqueous buffer and in human plasma-containing samples. Accordingly, iso-osmolar liposomes containing 52.8 mM calcein, 44.5 mM (0.26% w/v) NaCl, pH = 7.4, 0.292 osmol·kg^{-1} were prepared. To determine the effect of an osmotic gradient on leakage from liposomes at different plasma concentrations, lipid films were hydrated with a hypo-osmolar solution comprising 52.8 mM calcein in MilliQ, pH = 7.4, 0.215 osmol·kg^{-1}.

S2.2. Tranexamic acid-encapsulating liposome preparation and characterization

Settings used for photon correlation spectroscopy: 90 degrees measurement angle, count rate > 200 Kcps, unimodal analysis, refractive index dispersant = 1.331, viscosity = 0.8872, measurement

temperature = RT, real refractive index = 1.46, core real refractive index = 1.327, radius ratio = 0.94117, Mark-Houwinck parameters: A = 0.50, K = 0.000138, absorption (at 633 nm) = 0.0.

In the electrophoretic mobility determinations, the Zetasizer sample chamber was thoroughly rinsed with a solution containing 0.1% Triton X-100 (20 mL), ethanol (20 mL), and MilliQ water (20 mL) before each measurement. Gelfiltered LUVETs were diluted 5 × with physiological buffer (RT) to a 1-mM final phospholipid concentration in a 2.5-mL sample volume. The sample and physiological buffer were analyzed at RT. The zeta potential of LUVETs was corrected for the measured zeta potential of physiological buffer.

Settings used for differential scanning calorimetry: scan rate = 55 °C/h, filter period = 2 s, resting temperature = 15 °C, cell concentration = 3.0 mM, starting temperature check enabled, desiccation while thermostat, real-time baseline subtraction, and pre-scan waiting period = 300 s.

S2.3. Heat-induced tranexamic acid release from thermosensitive LUVETs in physiological buffer

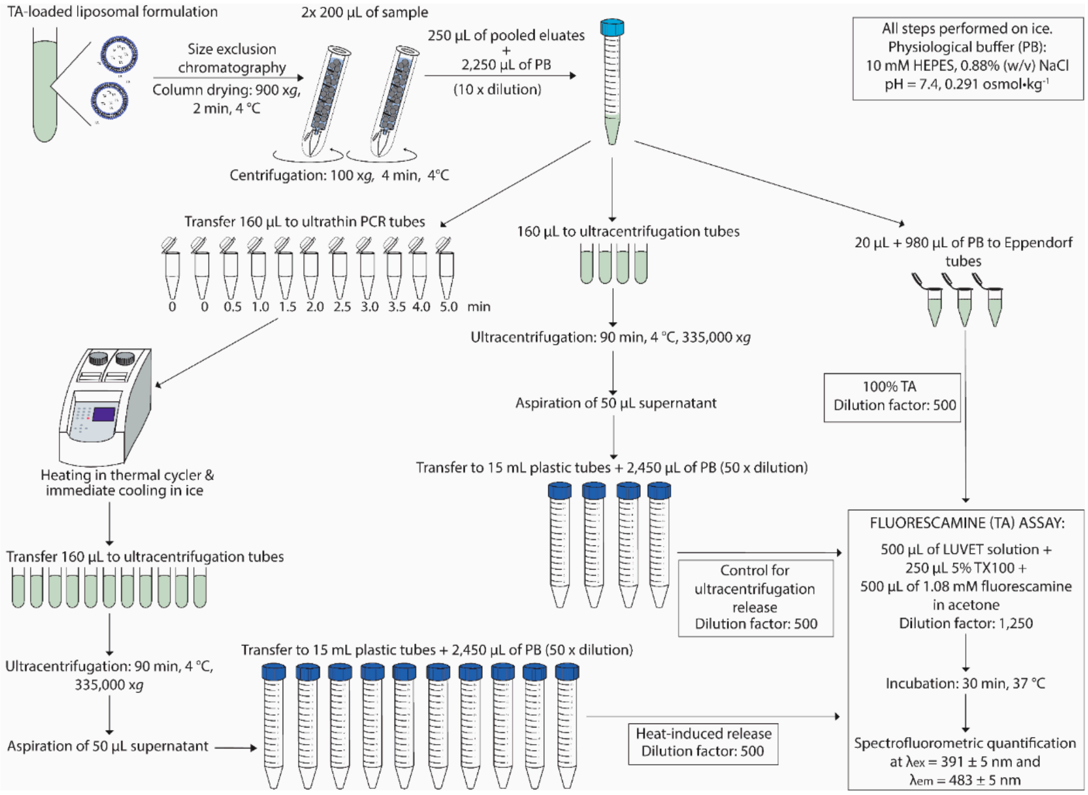

Figure S2. Experimental workflow related to the quantitative determination of heat-induced TA release from thermosensitive DPPC:DSPE-PEG (96:4) and DPPC:MPPC:DSPE-PEG (86:10:4) LUVETs. Schematics were adapted from [3].

S2.4. Animals and surgical procedures

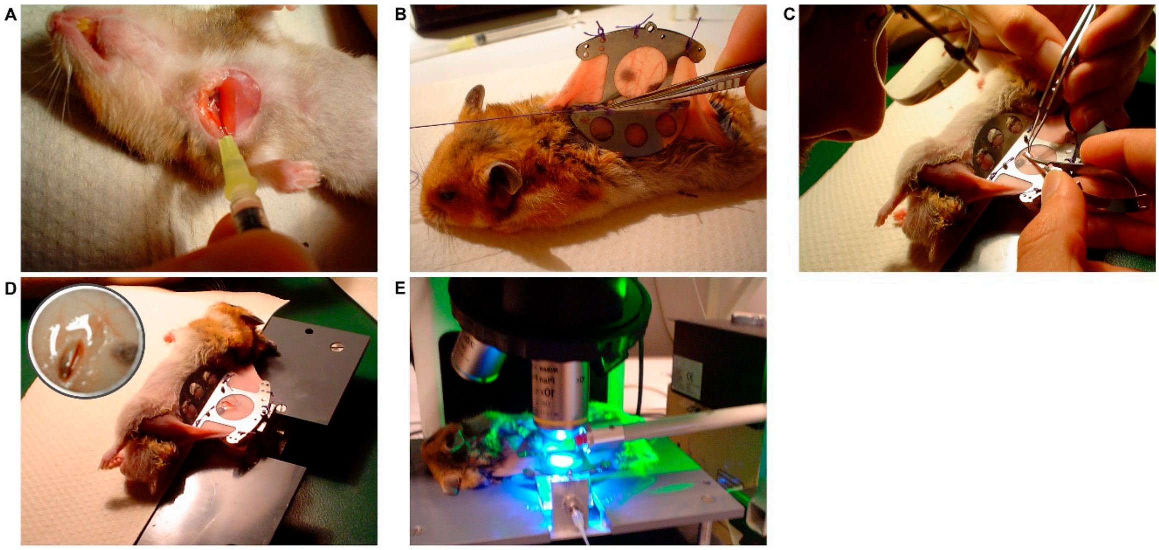

Figure S3. Key procedural steps in intravenously injecting solutions, affixing the optical chamber to the animal's dorsal skin. (A) The thoracic skin overlaying the jugular and subclavian vein was excised to enable the injection of solutions via the subclavian vein. The needle, bent to a ~60 degree angle, was inserted at a sharp angle from the flank and guided to the subclavian/jugular vein junction, where the solution was gently infused. The needle was retracted slowly along the thoracic surface such that the fat and fascia covering the subclavian vein would deter excessive bleeding. (B) The dorsal skin was shaved and, in this case, depilated to reveal the blood vessels as seen through the annular window of the optical chamber. The skin was loosely 'sandwiched' between two aluminum frames and secured with sutures looped through the small holes in the frames and through the skin. (C) The skin overlaying the blood vessel of interest was removed with microsurgical scissors. (D) The animal was secured to a microscope stage through pins that interlocked with the optical chamber. The open wound was kept moist with PBS (37 °C) to maintain quasi-physiological conditions for the target blood vessel (insert) and prevent muscle twitching due to desiccation. A robustly secured optical chamber, albeit ensuring that the interpositioned skin is not squeezed so as to not hamper blood flow, also reduces motion of the region of interest during intravital microscopic imaging, shown in (E). Some features in the images deviate from the actual protocol for purposes of illustration (anesthesia, syringes, microscope stage, microscope, and laser system). The protocol and images were adapted from [1].

S2.5. Intravital fluorescence microscopy and laser-induced thrombosis model

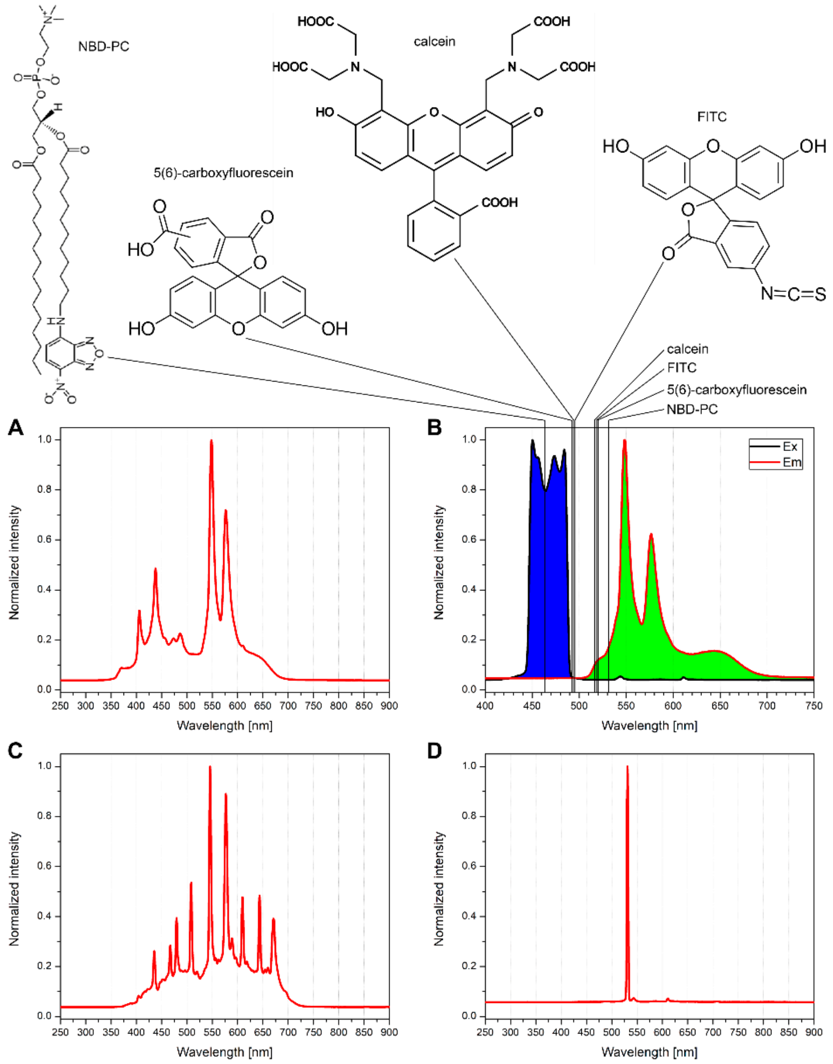

Figure S4. (A) Spectrum of emitted light by the Leica EL6000 light source. (B) The optical properties of the filter set used for intravital fluorescence microscopy. The transmission range of the excitation (ex) light filter is shown in blue (spectrum, black line), whereas the transmission range of the emission (em) filter is shown in green (spectrum, red line). The wavelengths at which NBD-PC, 5(6)-carboxyfluorescein, calcein, and FITC exhibit their absorption maximum and fluorescence emission maximum are demarcated to show that the filter set was appropriate to image all fluorophores. Al spectra were normalized to maximum intensity.

Table S2. Summary of experimental settings used in the in vivo study.

ANIMAL		LASER SETTINGS				IMAGING SETTINGS									
**F**	**Injection volume [μL]**	**Laser pulse [ms] (site)**	**N**	**No. of pulses**	**Pulse interval [ms]**	**Opt. zoom**	**Exp. time [ms]**	**Gain**	**Sat.**	**Gam-ma**	**Dia-phragm**	**Epi-ill. int. EL6000**	**Transill. int. Storz [%]**	**Imaging rate [fps]**	**Autom. shutter control**
1	-	60 (V1)<br>60 (V1)<br>120 (V1)<br>120 (V1)<br>120 (V1)	1	6<br>6<br>3<br>5<br>5	100<br>100<br>100<br>100<br>100	5.0	590	5.0 ×	1.40	1.43	100% open	5 (100%)	25	0.39	off
2	150	60 (V1)<br>60 (V2)<br>60 (V2)<br>60 (V2)	1	6<br>6<br>6<br>6	100<br>100<br>100<br>100	5.0	520	5.0 ×	1.40	1.43	100% open	5 (100%)	25	0.43	on
3	40	120 (V1)<br>180 (V2)	1	3<br>1	100<br>-	5.0	11.4	2.9 ×	1.40	1.43	100% open	5 (100%)	0	0.43	on
4	150	60 (V1)<br>120 (V2)<br>180 (V3)	1	3<br>3<br>3	2000<br>0.5<br>0.5	5.0	97.2	6.9 ×	1.40	0.44	100% open	5 (100%)	0	0.71	on
5	200<br>400	120 (V1)	2	3	100	5.0	920<br>920	5.0 ×	1.40	0.50<br>1.43	100% open	5 (100%)	0	0.29	on
6	400	1 (V1)<br>1 (V1)<br>1 (V1)<br>60 (V2)<br>10 (V3)<br>120 (V4)	1	60<br>60<br>120<br>6<br>10<br>1	2<br>2<br>1<br>100<br>500<br>-	5.0	719	5.0 ×	1.40	1.43	100% open	5 (100%)	25	0.34	on

Abbreviations: F, formulation; N, group size (number of hamsters); no., number; opt., optical; exp., exposure (CCD camera); sat., saturation; ill., illumination; fps, frames per second; autom., automated; V, venule site that was illuminated - each number represents a different site (upstream/downstream) on the same venule. F1: control group, no intravenous administration. F2: anti-CD62P monoclonal antibodies, label CD62P-expressing activated platelets in laser-induced thrombi [1]. F3: 49.6 mM calcein, 0.23% NaCl, pH = 7.4, 0.292 osmol·kg^{-1}. F4: 3.4 mM 5(6)-carboxyfluorescein in physiological buffer. F5: DSPC:DSPE-PEG (96:4) containing 20 μM 5(6)-carboxyfluorescein in physiological buffer. F6: DPPC:NBD-PC:DSPE-PEG (91:5:4) in physiological buffer.

The components of the custum-built laser system are shown in Figure S5. The laser beam was reflected by 2 adjustable 100% reflective mirrors (Thorlabs) into a light guide that could be rotated along its longitudinal axis to radially position the laser beam. A high-speed shutter (model Lambda SC *Smart*Shutter, Sutter Instrument, Novato, CA) was placed between the reflective mirrors to control the laser pulse width (via a model LB-SC controller and Sutter software). The terminal aperture of the laser guide contained a plano-convex lens (Thorlabs). A reflective mirror was fixed at an angle (135 degrees relative to the light path) into a linearly adjustable probe slid over the light guide to focus the laser onto the target blood vessel at approximately 45 degrees relative to the plane of the optical chamber. The focal point of the laser was situated at approximately 10 cm from the reflective mirror in the light guide tip, producing a slightly oval spot size of 2×10^{-3} mm^2.

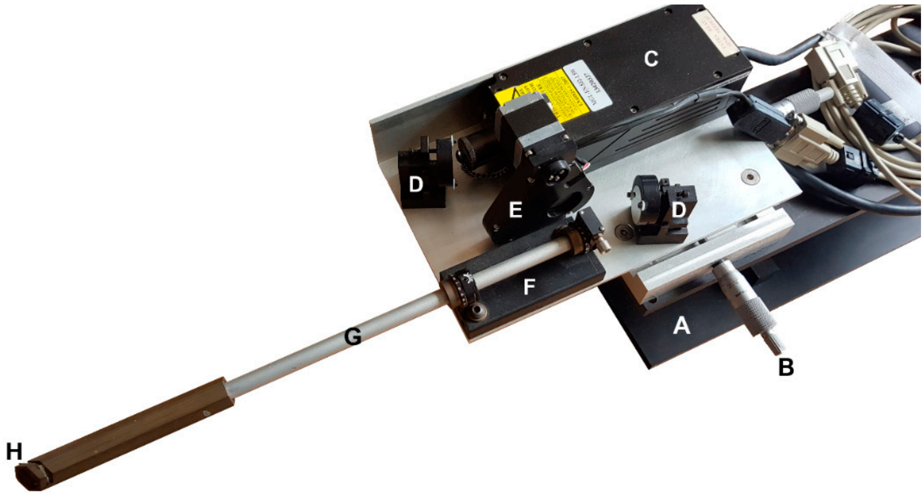

Figure S5. Custom-built laser system used to induce thrombosis in hamster dorsal venules during intravital fluorescence microscopy. (A) Sturdy steel base (9 kg), (B) XY translator stage, (C) CNI 532-nm tunable diode-pumped solid state laser, (D) adjustable reflective mirror, (E) Sutter Lambda SC *Smart*Shutter, (F) light guide holder and rotator, (G) light guide, (H) reflective mirror at the end of a converging lens-containing light guide segment.

S2.6. Image analysis

The isolated frames depicting the largest thrombus (group 1; section 2.9) or the most fluorescent thrombus (groups 2-6; section 2.9) were loaded into Photoshop and duplicated. The duplicated images were subjected to a green filter in the hue/saturation control panel as shown in Figure S6. The standard color range for the red, yellow, green, cyan, blue, and magenta hues spans 30° of the core hue + 30° into the neighboring hues on each end (Figure S6G). The lightness of all hues except for the green was completely dimmed to a value of -100. In order to retain the full bandwidth of the green hue, the long periphery of the yellow was cut off at 75° (Figure S6B) while the short periphery of the cyan hue was cut off at 165° (Figure S6D). The 165° and 75° values correspond to R/G/B coordinates 0/255/191 and 191/255/0, respectively. These RGB coordinates in turn translate to 496 nm and 559 nm, respectively (https://academo.org/demos/wavelength-to-colour-relationship/). Accordingly, only pixels that match these hue values were retained in the filtered duplicates, and hence show whether any of the fluorophores had been incorporated into the laser-induced thrombus (Figure S4).

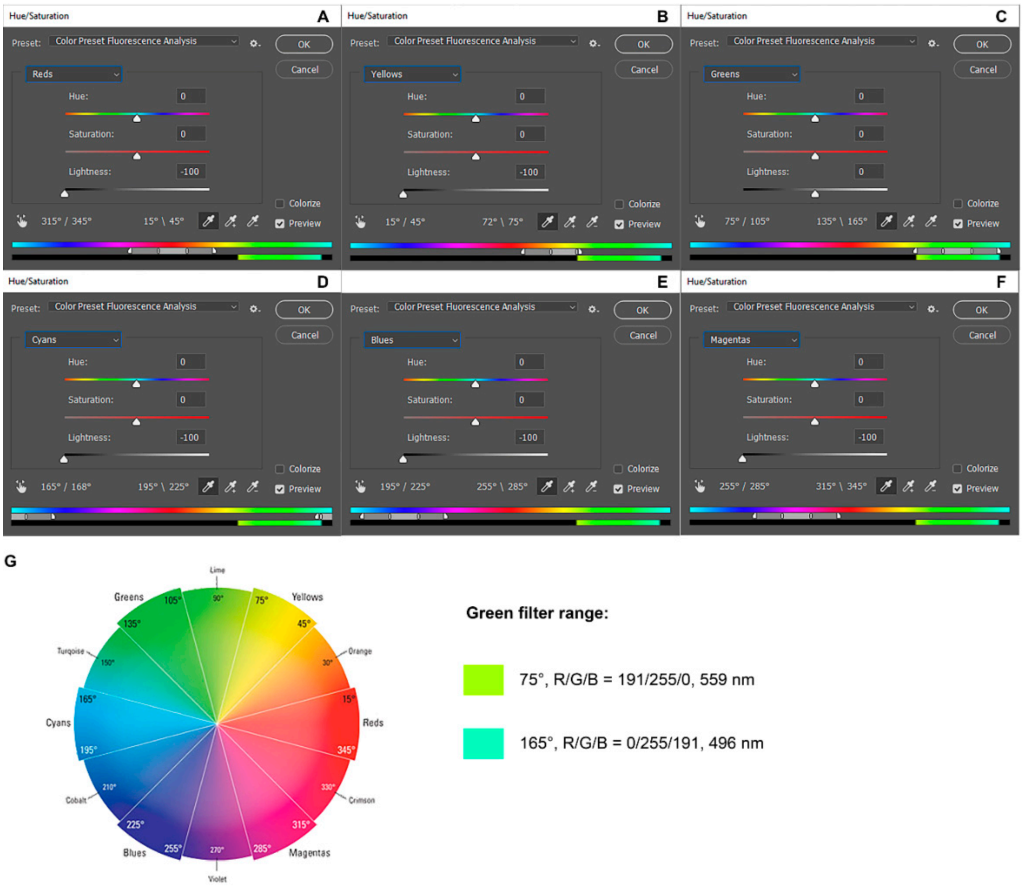

Figure S6. Saturation and lightness settings per hue used for the analysis of green fluorescence in laser-induced thrombi in Photoshop. The settings are delineated for red (A), yellow (B), green (C), cyan (D), blue (E), and magenta (F). The hue range of the yellow and cyan was cut off at 75° and at 165°, respectively (bottom spectral sliders in B and D, respectively), so as to allow both 30° peripherals of the green hue (G). The legend in (G) shows the corresponding RGB coordinates of the green hue boundaries as well as the corresponding wavelength. Consequently, the filtered duplicate fames showed only colors in the 496-559 nm wavelength range - i.e., the fluorescence emission range of calcein, CF, NBD, and FITC (Figure S4).

S3. Results

S3.1. Tranexamic acid-encapsulating DPPC:DSPE-PEG liposomes have the most favorable physicochemical properties for antifibrinolytic SSPLT

Table S3. Summary of physicochemical properties of liposomal formulations encapsulating different drugs.

Ref.	Formulation (molar ratio)	Drug loading technique	Encapsulant	LogP	Water solubility (mg/mL)	MW (g·mol^{-1})	Drug:lipid ratio (mol/mol)	$E_{eff.}$ (%)	Quantitation method E_{eff}
[4]	DPPC:HSPC:chol:DSPE-PEG1900:DSPE-Rh (100:50:30:6:1.2)	Hydration lipid film	Calcein	-3.1	NL	622.5	0.06[1]	NL	Gel filtration to remove free drug. Encapsulated drug and lipids (phosphate assay) were determined to calculate drug:lipid ratio.
[5]	DSPC:DOPE:CHEMS (13:1:10)	Hydration lipid film	Calcein	-3.1	NL	622.5	0.66[2]	66.5	Centrifugal-ultrafiltration method to quantitate free drug. Compared to added drug.
[6]	EPC:chol (60:40)	Hydration lipid film	Cytarabine	-2.1	43.8	243.2	0.05	85.2	Ultracentrifugation. After digestion, encapsulated drug content was determined and compared to the amount of added drug.
[7]	DSPC:DSPG:chol (7:2:1)	Heated passive encapsulation / Cu^{2+} gradient	Cytarabine / daunorubicin	-2.1 / 1.8	43.8 / 39.2	243.2 / 527.5	0.5 / 0.1	5 / 100	Gel filtration after loading of cytarabine. Lipid and cytarabine content was determined in the eluents. Free daunorubicine was removed by tangential flow chromatography. The formula for $E_{eff.}$ was not reported.
[8]	DPPC:DMPC:DSPC (4:1:1)	Reverse-phase evaporation	Gemcitabine	-1.5	22.3	263.2	NL	30	Removal of free gemcitabine by gel chromatography. $E_{eff.}$ was determined by the Bligh and Dyer method, unclear whether lipid content was determined (encapsulated drug = added drug - drug in supernatant).
[9]	HSPC:chol:DSPE-PEG2000	Reverse-phase evaporation	Gemcitabine, Fe_3O_4	-1.5	22.3	263.2	NL	87.2	Centrifugation and dialysis. Free and total drug was evaluated. Final lipid concentration is unknown.
[10]	DSPC:chol (3:2)	Hydration lipid film,* ammonium sulfate gradient[#]	Topotecan	0.5	1	421.4	6.9×10^{-3},* 0.19[#]	5.3,* 93.1[#]	Extraliposomal ammonium salt and free topotecan was removed by gel filtration. Phosphate assay and spectrophotometry was performed to determine the lipid and

									drug content, respectively. The formula for $E_{eff.}$ was not reported.
[11]	DSPC:chol:DSPG:DSPE-PEG2000 in mole ratios:<br>90:45:10:0 (initial drug lipid:ratio 1:60)<br>70:35:30:10.2 (initial drug lipid:ratio 1:60)<br>70:35:30:0 (initial drug lipid:ratio 1:30)<br>90:45:10:10.9 (initial drug lipid:ratio 1:30)<br>90:90:10:0 (initial drug lipid:ratio 1:30)<br>70:70:30:0 (initial drug lipid:ratio 1:60)<br>90:90:10:14.3 (initial drug lipid:ratio 1:60)<br>70:70:30:12.8 (initial drug lipid:ratio 1:30)<br>70:70:30:0 (initial drug lipid:ratio 1:30)	Hydration lipid film	Topotecan	0.5	1	421.4	1.48[2,3]<br>0.94[2,3]<br>2.00[2,3]<br>0.61[2,3]<br>1.10[2,3]<br>1.07[2,3]<br>0.09[2,3]<br>1.92[2,3]<br>1.82[2,3]	9.96<br>7.27<br>6.70<br>2.36<br>3.76<br>7.61<br>0.68<br>11.44<br>6.21	Dialysis was performed to remove free drug. HPLC to determine drug content in liposomes; this was compared to the quantity of drug added.
[12]	HSPC:chol:DSPG (2:1:0.8)	Supercritical fluid of carbon dioxide,*<br>hydration lipid film#	Amphotericin B	0.8	0.08	924.1	NL	> 95,*<br>~90#	Ultracentrifugal separation. HPLC to determine free and encapsulated drug.
[13]	DPPC:chol:DSPE-PEG2000 (62.7:31.3:6)	Hydration lipid film	Amphotericin B	0.8	0.08	924.1	0.06[4]	10.1	Removal of free drug by centrifugation. Phospholipid and drug content determined (not specified in what samples).
[14]	SPC	Hydration lipid film	Amphotericin B	0.8	0.08	924.1	0.02[5]	24	Removal of free drug by centrifugation. Liposomes were ruptured by sonication. Encapsulated drug was determined by spectrophotometry. $E_{eff.}$ was calculated by comparing liposomal drug to the initially added drug.
[15]	DPPC:DSPC:DSPE-PEG2000 (80:15:5)<br>DPPC:MSPC:DSPE-PEG2000 (85:10:5)	pH gradient	Doxorubicin	1.3	1.18	543.5	0.13<br>0.05	87<br>100	Centrifugal separation. Phospholipid assay. Encapsulated drug was determined by spectrofluorometry. Drug:lipid ratio after formation was compared to the initial drug:lipid ratio.

[16]	DPPC:DSPC:GM_1 (90:10:3-9)	pH gradient	Doxorubicin	1.3	1.18	543.5	0.06[6]	95-98	All liposomes were gel filtered. Encapsulated drug was determined by a fluorescence spectrometer. Lipids not determined.
[17]	DPPC:Brij78 (96:4)	pH gradient	Doxorubicin	1.3	1.18	543.5	0.04[6]	95	Gel filtration to remove unencapsulated drug. Fiske and Subbarow phosphate assay and fluorescence spectrometry for drug content. $E_{eff.}$ = final drug:lipid ratio/initial drug:lipid ratio.
[18]	DPPC:chol (80:20)	Ammonium sulfate gradient	Doxorubicin, Fe_3O_4	1.3	1.18	543.5	NL	90.9	Removal of free drug by dialysis. The concentration of drug in supernatant was determined by spectrophotometry. The $E_{eff.}$ was calculated with the following formula: E_{eff} (%) = (W_{int}- W_{sup})/W_{int}×100%, where W_{int} and W_{sup} represent the weight of drug added initially and amount of drug in supernatant.
[19]	SPC:chol (9:6)<br>SPC:chol:DSPE-PEG2000 (9:6:0.075)<br>SPC:chol:DSPE-mPEG2000 (9:6:0.75)<br>SPC:chol:DSPE-mPEG2000:DSPE-PEG2000 (9:6:0.75:0.075)	Hydration lipid film	Paclitaxel	2.5	0.006	853.9	0.058<br>0.059<br>0.058<br>0.056	86.8<br>88.7<br>87.9<br>88.1	Removal of free drug by centrifugation. Sonication to rupture liposomes and determine encapsulated drug by HPLC. This was compared to the drug added during preparation.
[20]	SPC:chol (90:10)<br>SPC:chol:DSPE-PEG (90:10:5)	Hydration lipid film	Paclitaxel	2.5	0.006	853.9	$6.9\times(10^{-3})$[3,5,7]<br>$4.8\times(10^{-3})$[3,5,7]	61<br>57	Free drug removal by centrifugation. Encapsulated drug was determined by HPLC and compared to the quantity of drug added during preparation.
[21]	EPC:chol (55:45)	pH gradient	Vincristine (sulfate)	2.8	0.03	923	0.12[2,3,8]	> 95[a]	Gel filtration. Concentration of vincristine and lipid determined (according to Fiske and Subbarow) by column chromatography and spectroscopy. Formula for "trapping efficiencies" was not reported.
[21]	DSPC:chol (55:45)	pH gradient	Vincristine (sulfate)	2.8	0.03	923	0.17[2,3,8]	17[a]	Gel filtration. Concentration of vincristine and lipid determined (according to Fiske and Subbarow) by column chromatography and spectroscopy.

									Formula for "trapping efficiencies" was not reported.
[21]	DSPC:chol (55:45)	pH gradient	Vincristine (sulfate)	2.8	0.03	923	0.17[2,3,8]	> 98[b]	Gel filtration. Concentration of vincristine and lipid determined (according to Fiske and Subbarow) by column chromatography and spectroscopy. Formula for "trapping efficiencies" was not reported.
[22]	Chit-EPC	Hydration lipid film	Ketoprofen	3.1	0.051	254.3	9.0[9]	95	Dialysis. Drug content determined by HPLC. No further details reported.
[23]	PC:chol (60:40)	Reverse-phase evaporation*, extrusion technique#	Ketoprofen (conjugated to HPβCyd)	3.1	0.051	254.3	0.22[10]	61.6*, 54.8#	Spectrophotometric measurement of free drug that was separated by dialysis. Compared to the quantity of added drug during preparation.

Abbreviations (in order of appearance): MW, molecular weight; $E_{eff.}$, encapsulation efficiency; DPPC, 1,2-dipalmitoyl-sn-glycero-3-phosphocholine; HSPC, hydrogenated soy phosphatidylcholine; chol, cholesterol; DSPE, 1,2-distearoyl-sn-glycero-3-phosphoethanolamine; PEG1900/2000, polyethylene glycol with a MW of 1900/2000 $g·mol^{-1}$; DSPE-Rh, N-(lissamine Rhodamine B sulfonyl)-1,2-distearoyl-sn-glycero-3-phosphatidylethanolamine; NL, not listed; DSPC, 1,2-distearoyl-sn-glycero-3-phosphatidylcholine; DOPE, 1,2-dioleoyl-sn-glycero-3-phosphoethanolamine; CHEMS, cholesteryl hemisuccinate; EPC, egg phosphatidylcholine (L-α-phosphatidylcholine); DSPG, 1,2-distearoyl-sn-glycero-3-phosphoglycerol; DMPC, 1,2-dimyristoyl-sn-glycero-3-phosphocholine; HPLC, high-performance liquid chromatography; SPC, soy phosphatidylcholine (L-α-phosphatidylcholine); GM_1, ganglioside M1; Brij 78, polyoxyethylene (~20 units, MW ~880 g/mol) stearyl ether, 4 mol%, a surfactant; mPEG, methoxy polyethylene glycol; chit, chitosan (310 $kg·mol^{-1}$), a sterically-stabilizing polysaccharide; PC, phosphatidylcholine (L-α-phosphatidylcholine); HPβCyd, hydroxypropyl-β-cyclodextrin, an oligosaccharide used to improve aqueous solubility. Where applicable, drug:lipid ratios were calculated based on a [1]calcein MW of 662.54 $g·mol^{-1}$, a [2]DSPC MW of 790.16 $g·mol^{-1}$, a [2]CHEMS MW of 486.74 $g·mol^{-1}$, a [2]DOPE MW of 744.03 $g·mol^{-1}$, a [3]topotecan MW of 457.92 $g·mol^{-1}$, a [3]cholesterol MW of 386.7 $g·mol^{-1}$, [3]DSPG MW of 744.96 $g·mol^{-1}$, a [3]DSPE-PEG2000 MW of 2805.5 $g·mol^{-1}$, a [4]DPPC MW of 734.04 $g·mol^{-1}$, an [5]SPC MW of 758.07 $g·mol^{-1}$, an [5]amphotericin B MW of 924.1 $g·mol^{-1}$, a [6]doxorubicin MW of 543.53 $g·mol^{-1}$, a [6]GM_1 MW of 1546.8 $g·mol^{-1}$, a [7]paclitaxel MW of 853.90 $g·mol^{-1}$, a [8]vincristine MW of 923.04 $g·mol^{-1}$, an [8]EPC MW of 760.1 $g·mol^{-1}$, an [9]HSPC MW of 762.1 $g·mol^{-1}$, and a [10]PC MW of 760.1 $g·mol^{-1}$. The * and # indicate the corresponding $E_{eff.}$ and/or drug:lipid ratio when multiple loading techniques were applied in one study. [a]1-h incubation at 21 °C. [b]10-min incubation at 60 °C.

The low TA:lipid ratio in our formulations, compared to the E_{eff}s reported for other drugs in Table S3, stems from the fact that we deliberately chose to add 316 mmoles of TA to 5 mmoles of lipid, and is further accounted for by the liposome preparation method. The high-drug-low-lipid proportions were deliberate so that 1) the intraliposomal environment would be iso-osmolar relative to physiological conditions, and 2) only a small number of liposomes would have to be targeted to laser-induced thrombi to account for a pharmacologically relevant anti-fibinolytic milieu in laser-illuminated PWS vessels. These rationales were explained and demonstrated in [3]. When one adds > 60 × more hydrophilic drug molecules than lipids to a solution where only a tiny fraction of the volume will be occupied by the aqueous compartment of the formed liposomes, most of the water-dissolved TA will reside in the extraliposomal fraction. This TA-containing fraction is cleared in the size exclusion chromatography step(s), whereby only intraliposomal TA is retained in the eluate, and replaced by TA-lacking physiological buffer that the Sephadex G50 matrix was equilibrated in. Consequently, the ultimate TA content is profoundly reduced by the Sephadex matrix exchange while the lipid content stays more or less the same.

S3.2. Tranexamic acid-encapsulating PEGylated thermosensitive liposomes release content at temperatures equal to and above the phase transition temperature in buffered solution

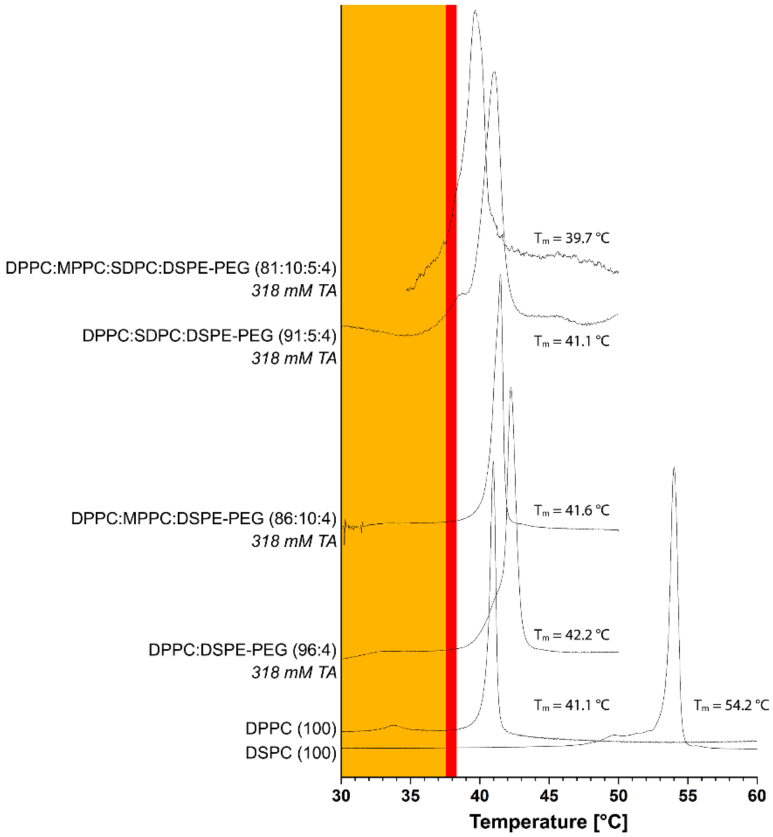

Figure S7. Differential scanning calorimetry thermograms of TA-encapsulating LUVETs of different phospholipid compositions (from bottom to top, formulations 3, 6, 14, and 19) in physiological buffer compared to empty DPPC and DSPC liposomes in water. The phase transition temperature (T_m) is indicated in the warm temperature shoulder of each thermogram. The orange region designates temperatures that the LUVETs will be exposed after intravenous administration (up to the normal body temperature; 36.5-37.5 °C). The red temperature zone represents the range corresponding to the onset of fever (> 37.5 °C or 38.3 °C).

S3.3. Plasma stabilizes thermosensitive liposomes and reduces the extent of leakage at body temperature

Figure S8 demonstrates that calcein leakage from DPPC:DSPE-PEG (96:4) and DPPC:MPPC:DSPE-PEG (86:10:4) LUVETs does not occur at 4 °C regardless of osmotic differences between the intra- and extraliposomal space.

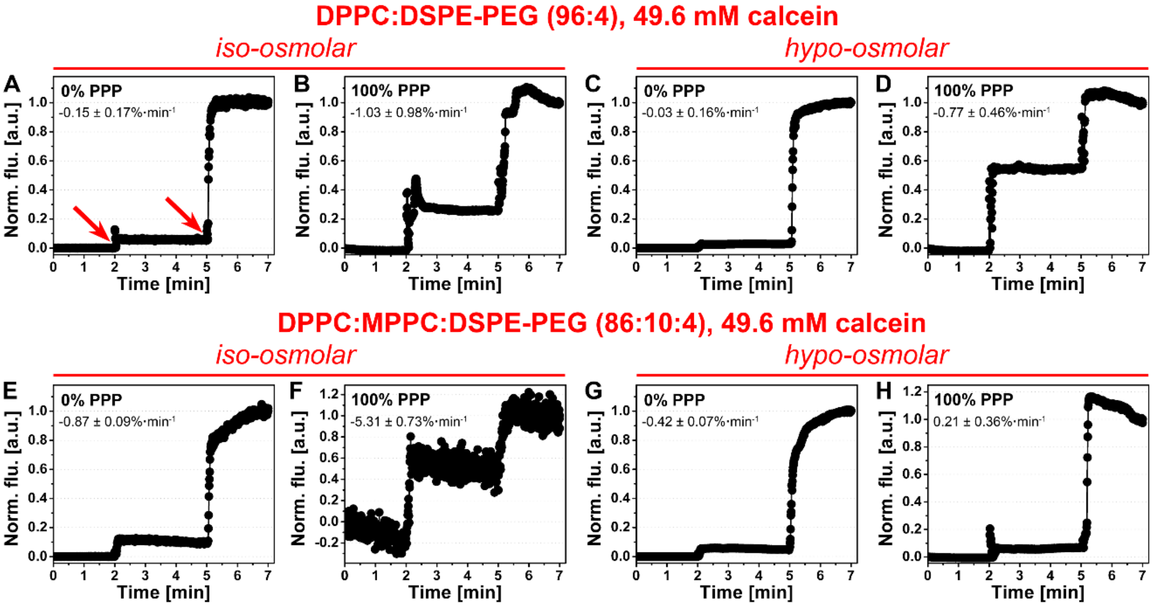

Figure S8. Calcein leakage at 4 °C from DPPC:DSPE-PEG (96:4) LUVETs containing calcein at iso-osmolar (A, B) and hypo-osmolar (C, D) conditions and from DPPC:MPPC:DSPE-PEG (86:10:4) LUVETs containing calcein at iso-osmolar (E, F) and hypo-osmolar (G, H) conditions relative to physiological buffer and plasma. Representative leakage kinetics are plotted as a function of time of LUVETs suspended in physiological buffer without platelet-poor plasma (0% PPP; A, C, E, G) and of LUVETs added to 100% PPP (B, D, F, H). The left red arrow in (A) indicates the time point at which LUVETs (15 μL) were added to the cuvette containing 1485 μL of physiological buffer and/or PPP, while the right red arrow indicates the addition of Triton X-100 to induce 100% calcein release and unquenched fluorescence.

The stability of DPPC:DSPE-PEG (96:4) and DPPC:MPPC:DSPE-PEG (86:10:4) LUVETs containing a hypo-osmolar, self-quenching calcein concentration (49.6 mM calcein in MilliQ, pH = 7.4, 0.210 osmol·kg^{-1}) was assessed in physiological buffer containing increasing platelet-poor plasma (PPP) concentrations and PPP only. The DPPC:DSPE-PEG (96:4) and DPPC:MPPC:DSPE-PEG (86:10:4) LUVETs, prepared with a hypo-osmolar calcein solution relative to physiological buffer and plasma, had a mean ± SD diameter of 130.4 ± 1.6 nm and 162.8 ± 3.5 nm, respectively, and a mean ± SD polydispersity index of 0.270 ± 0.067 and 0.077 ± 0.022, respectively. The passive leakage rates and kinetics at 37 °C are plotted in Figure S9.

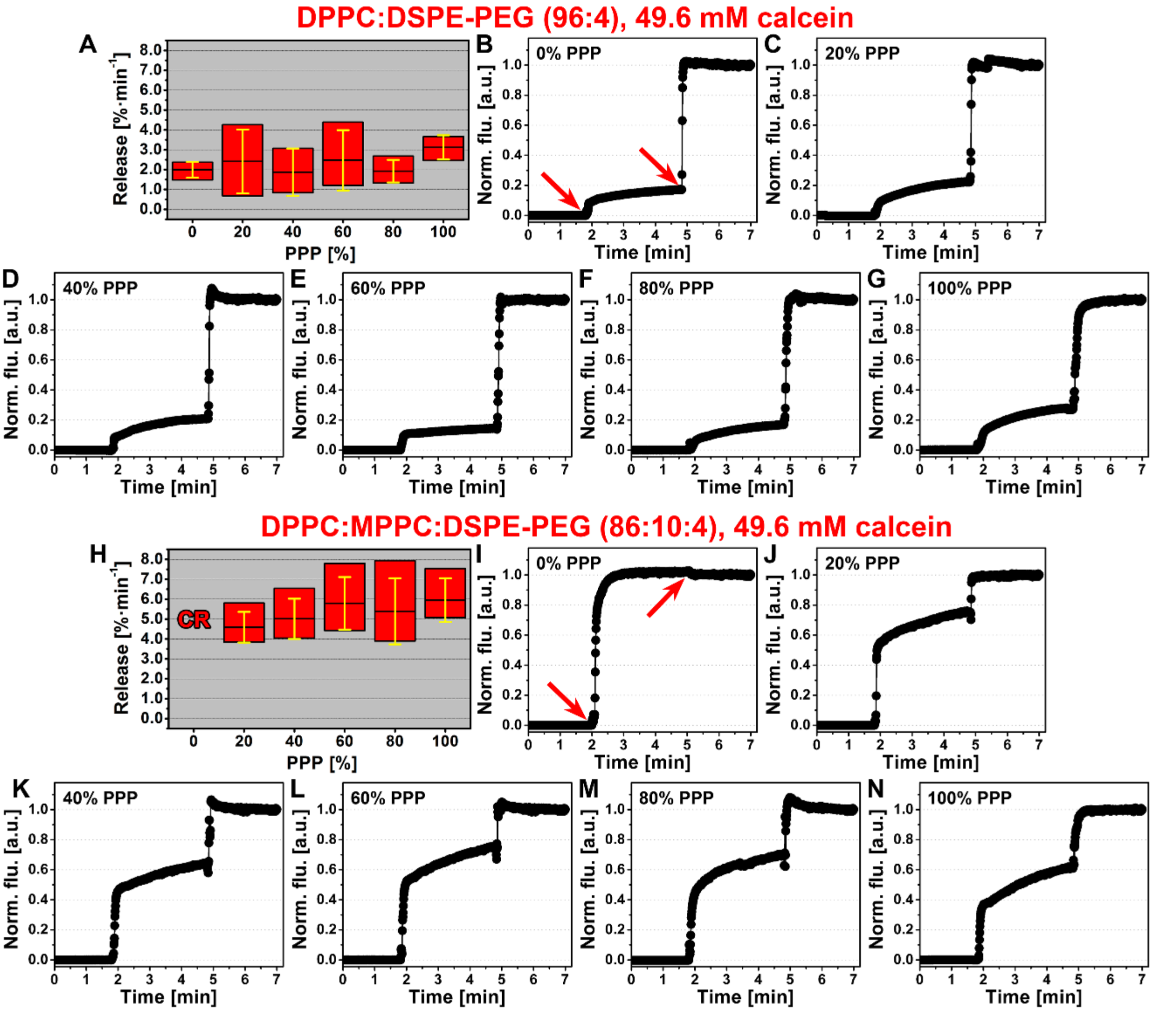

Figure S9. Calcein leakage at body temperature (37 °C) from DPPC:DSPE-PEG (96:4) LUVETs (A-G) and DPPC:MPPC:DSPE-PEG (86:10:4) LUVETs (H-N) that had been loaded with a hypo-osmolar calcein solution (0.210 osmol·kg-1) relative to physiological buffer and plasma (0.293 osmol·kg-1). Leakage rates were plotted as a function of PPP concentration (A, H) in modified box-whisker plots showing the maximum, minimum, mean, and SD release rate (N = 4 or 5 per group). The 'CR' in (H) stands for 'complete release.' Representative leakage kinetics are plotted vs. time at increasing platelet-poor plasma (PPP) concentrations, indicated in the upper left corner (B-G and I-N). The left red arrow in (B and I) indicates the time point at which LUVETs (15 μL) were added to the cuvette containing 1485 μL of physiological buffer and/or PPP, while the right red arrow indicates the addition of Triton X-100 to induce 100% calcein release and unquenched fluorescence.

DPPC:DSPE-PEG LUVETs exhibited comparable calcein leakage rates irrespective of the PPP concentration, which were significantly lower than for DPPC:MPPC:DSPE-PEG LUVETs at 20%, 40%, 60%, and 80% PPP. When DPPC:MPPC:DSPE-PEG LUVETs were added to equilibration buffer, 100% calcein leakage occurred within 1 min. The addition of PPP stabilized passive calcein leakage to some extent. The high relative fluorescence of the LUVETs following addition to PPP-containing solution suggests a biphasic process of initially rapid leakage (few seconds) followed by slower leakage. It should be noted that the plotted leakage rates do not reflect the rapid leakage phase since the leakage rates were measured between roughly 3-5 min. Given the fact that DPPC:MPPC:DSPE-PEG LUVETs containing an iso-osmolar calcein concentration had similar physicochemical properties but lower fluorescence following addition to the PPP-containing solutions, it is proposed that the initial rapid leakage of calcein from hypo-osmolar DPPC:MPPC:DSPE-PEG LUVETs was due to the osmolarity gradient.

Finally, calcein release DPPC:DSPE-PEG (96:4) and DPPC:MPPC:DSPE-PEG (86:10:4) LUVETs containing a hypo-osmolar, self-quenching calcein concentration (49.6 mM calcein in MilliQ, pH = 7.4, 0.210 osmol·kg^{-1}) was assessed during a 20 °C → 47.0 °C temperature ramp in a repurposed Roche LightCycler 480 II system. These experiments were performed to prove the importance of an osmotic balance between the intra- and extraliposomal space. As shown in Figure S10, the difference of 0.083 osmol·kg^{-1} was sufficient to induce membrane perturbations and content leakage at temperatures of > 20 °C below T_m. DPPC:DSPE-PEG (96:4) LUVETs exhibited slower release than DPPC:MPPC:DSPE-PEG (86:10:4) LUVETs in the presence of PPP. The rate of calcein release was decelerated by plasma in a proportional manner at temperatures around 20 °C - a phenomenon that prevailed in case of DPPC:DSPE-PEG (96:4) LUVETs but not DPPC:MPPC:DSPE-PEG (86:10:4) LUVETs during the temperature ramp. Upon reaching body temperature, DPPC:DSPE-PEG (96:4) LUVETs had released 65.5 ± 11.9%, 33.2 ± 8.2%, 21.9 ± 2.8%, and 16.6 ± 2.6% of calcein in physiological buffer (0% PPP), 20% PPP, 60% PPP, and 100% PPP, respectively. DPPC:MPPC:DSPE-PEG (86:10:4) LUVETs had released 73.9 ± 13.1%, 92.4 ± 1.7%, 78.9 ± 16.0%, and 50.9 ± 5.8% of calcein in physiological buffer (0% PPP), 20% PPP, 60% PPP, and 100% PPP, respectively.

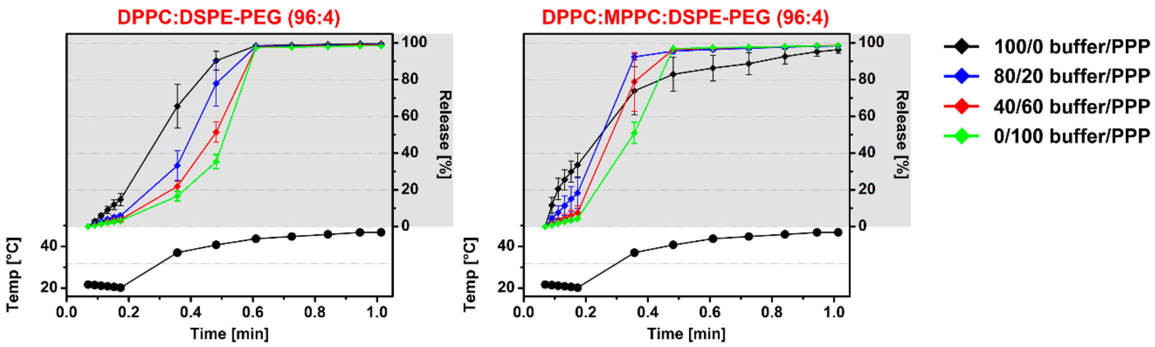

Figure S10. Calcein leakage during a 20 °C → 47.0 °C temperature ramp from DPPC:DSPE-PEG (96:4) LUVETs (A-G) and DPPC:MPPC:DSPE-PEG (86:10:4) LUVETs (H-N) that had been loaded with a hypo-osmolar calcein solution (0.210 osmol·kg^{-1}) relative to physiological buffer and plasma (0.293 osmol·kg^{-1}). The experiments (N = 8 per group, legend top right) were performed in a Roche LightCycler 480 II instrument. The temperature is plotted at the bottom (white) part of the figure and designated on the left *y*-axis. The percentage calcein release was derived from the fluorescence data and is presented in the top (gray) part of the figure and designated on the right *y*-axis. The temperature and fluorescence data points are matched. Fluorescence data are plotted as mean ± SD.

S3.4. LUVETs associate with hamster and human platelets

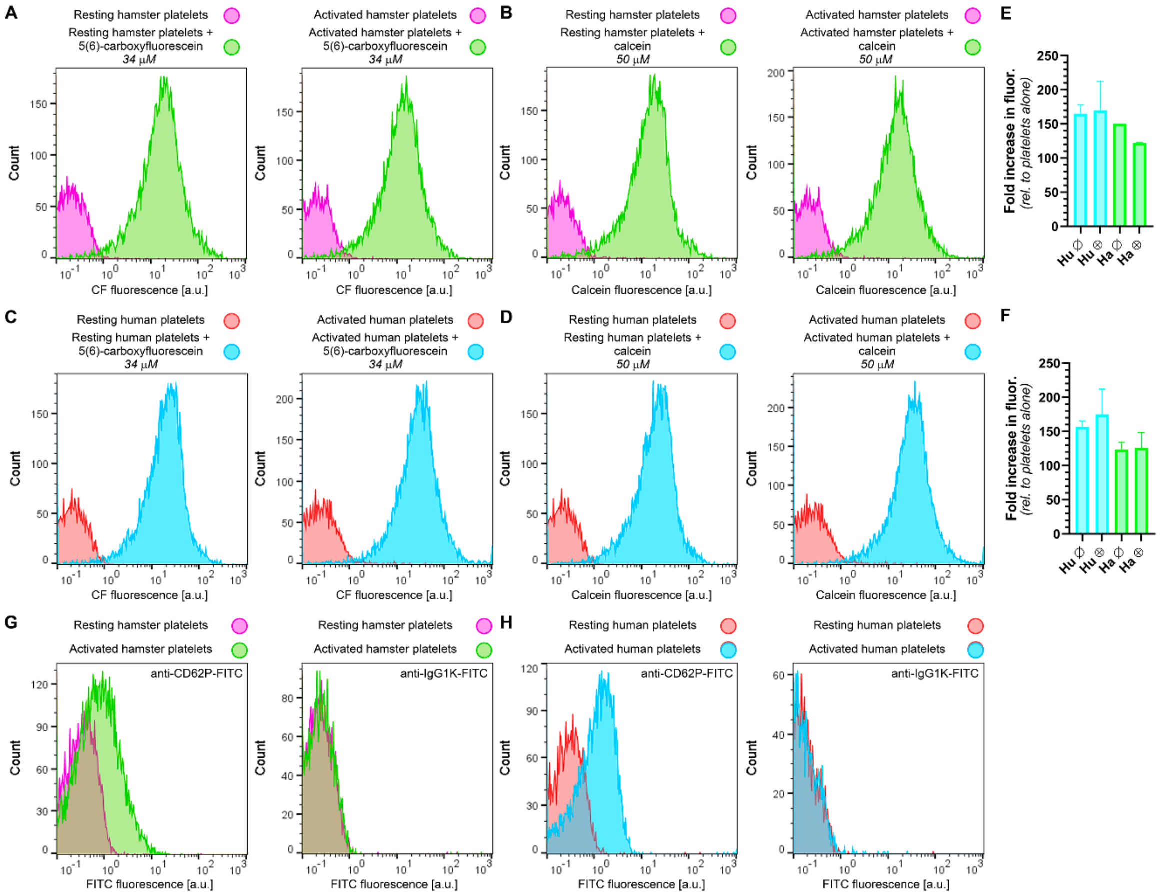

Figure S11. Representative flow cytograms of resting and convulxin-activated hamster platelets incubated with (A) 34 µM 5(6)-carboxyfluorescein (CF) and (B) 50 µM calcein (green traces). In (C) and (D), flow cytograms are shown of resting and convulxin-activated human platelets that had been incubated with the same concentration CF and calcein, respectively (blue traces). The purple (A, B) and red traces (C, D) represent fluorescence histograms of platelets that had not been incubated with fluorophores (control). The fold increase in fluorescence relative to non-labeled platelets in provided in (E) for CF and (F) for calcein (Hu, human; Ha, hamster; ∅, resting platelets; ⊕, activated platelets) (N = 2 per group). The extent of platelet staining with activation-dependent anti-CD62P-FITC antibodies and their respective anti-$IgG_{1\kappa}$-FITC isotype control antibodies is present in (G) for hamster platelets and in (H) for human platelets (N = 2 per group).

S3.5. LUVETs do not incorporate into laser-induced thrombi in vivo

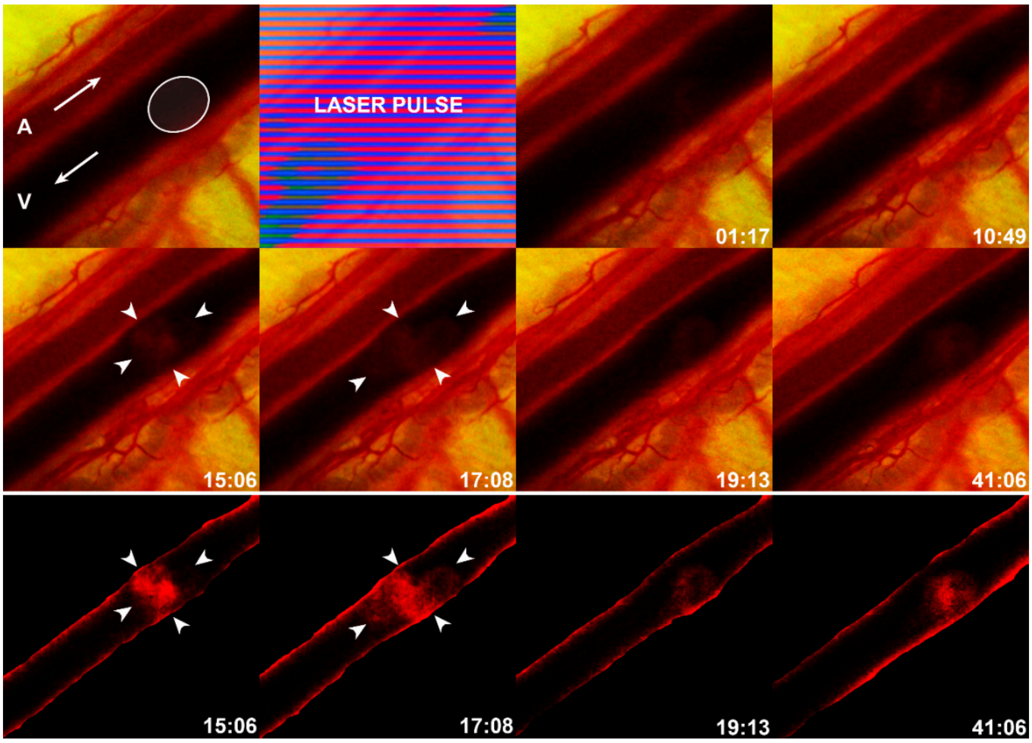

Figure S12. Thrombus kinetics in the negative control group (no fluorescent thrombus labeling) following multiple laser pulses (Table S2) administered to the encircled region in the top left panel. The arrows indicate the direction of blood flow in the venule (V) and arteriole (A). After illumination, a thrombus began to form and expand in time, indicated as minutes:seconds relative to the last laser pulse in the bottom right corner. The thrombus was visible as a white patch (arrowheads), resulting from the absence of light absorbers in the clot compared to blood, which comprises erythrocytes that contain the strongly light-absorbing chromophore hemoglobin. Consequently, the blood vessels always appear as dark columns regardless of the type of microscope illumination (epi- and transillumination). The panels in the bottom row (15:06 - 41:06) were duplicated and edited in Photoshop to reveal the laser-induced thrombus. The vessel segment was layered via cut after manual contouring and the background in the source image was blackened. The layered vessel segment was augmented in brightness (150%) and contrast (200%) to show the thrombus, which was visible due to reflection of incident light from the EL6000 light source and heating lamp.

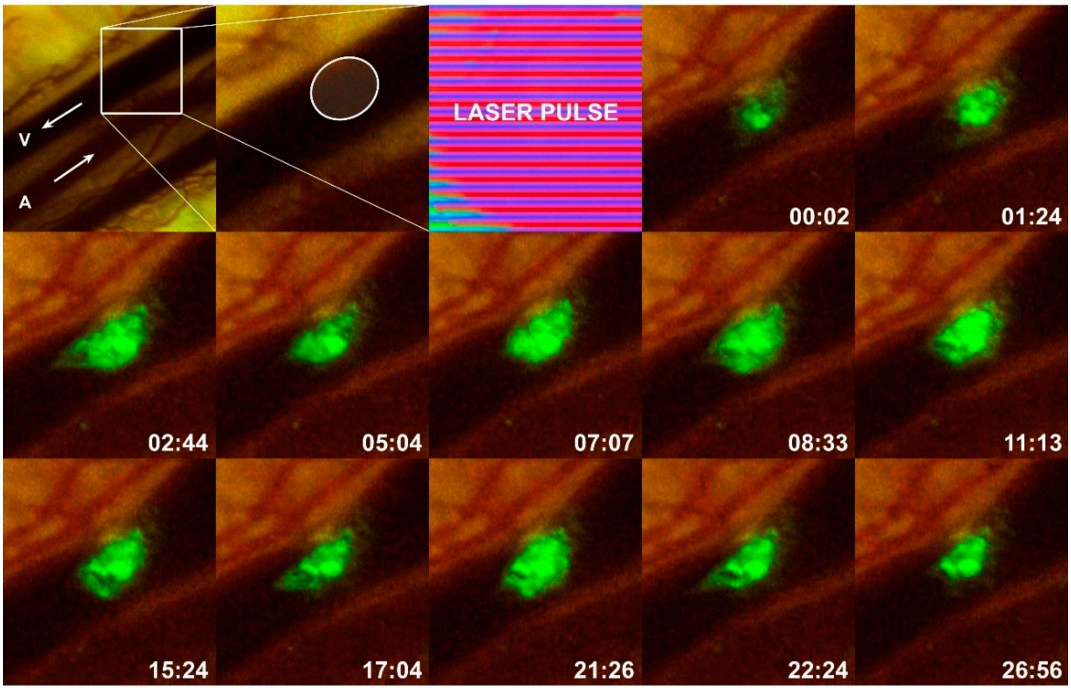

Figure S13. Time lapse series of a laser-induced thrombus intravitally stained with FITC-conjugated antibodies against CD62P (P-selectin), which is expressed only on the outer membrane of activated platelets that participate in clot formation. The arteriole (A) and venule (V) are indicated along with the direction of blood flow (arrows) in the top left panel. The quadrant represents the region of interest in the subsequent panels, with the circle delineating the site of laser illumination. The time after the last laser pulse is provided as minutes:seconds.

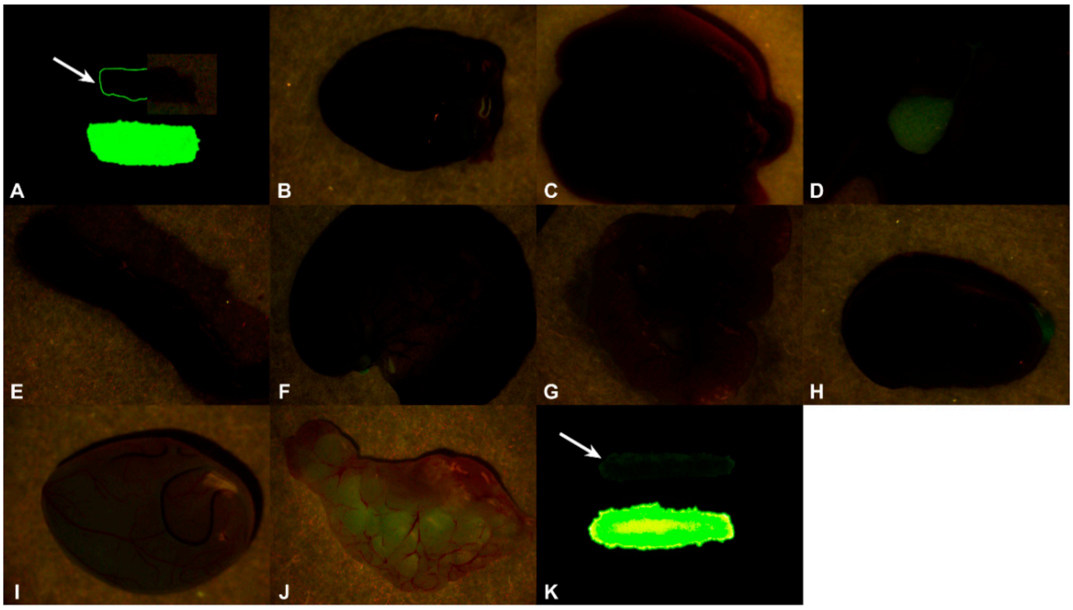

Figure S14. Fluorescence of FITC-conjugated anti-CD62P antibodies in (A) blood (arrow) and antibody solution (intense green smear), (B) heart, (C) lungs, (D) liver and gallbladder, (E) spleen, (F) stomach, (G) intestines, (H) kidney, (I) testicle, (J) epididymis, and (K) urine (arrow) and antibody solution (intense green smear). Half of the blood smear in (A) was enhanced by augmenting the intensity by 375% to demonstrate the lack of FITC fluorescence in blood. The other half of the smear was contoured. Organs were harvested approximately 90 min after antibody infusion and imaged using an exposure time of 1.5 s and a gain of 5.0 ×.

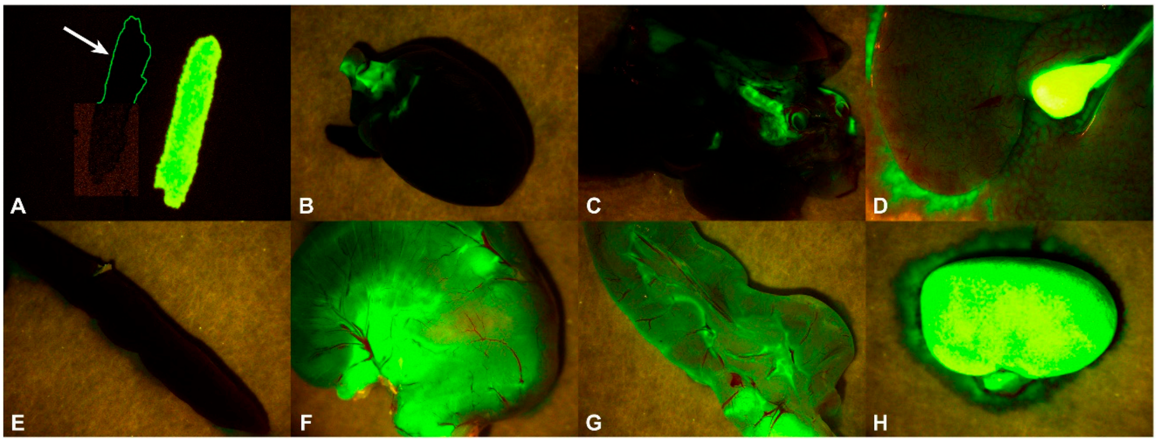

Figure S15. Fluorescence of 5(6)-carboxyfluorescein (CF) in (A) blood (arrow) and CF solution (intense green smear), (B) heart, (C) lungs, (D) liver and gallbladder, (E) spleen, (F) stomach, (G) intestines, and (H) kidney. Half of the blood smear in (A) was enhanced by augmenting the intensity by 150% to demonstrate the lack of CF fluorescence in blood. The other half of the smear was contoured. Organs were harvested approximately 85 min after 5(6)-carboxyfluorescein infusion and imaged using an exposure time of 1.6 s and a gain of 5.0 ×.

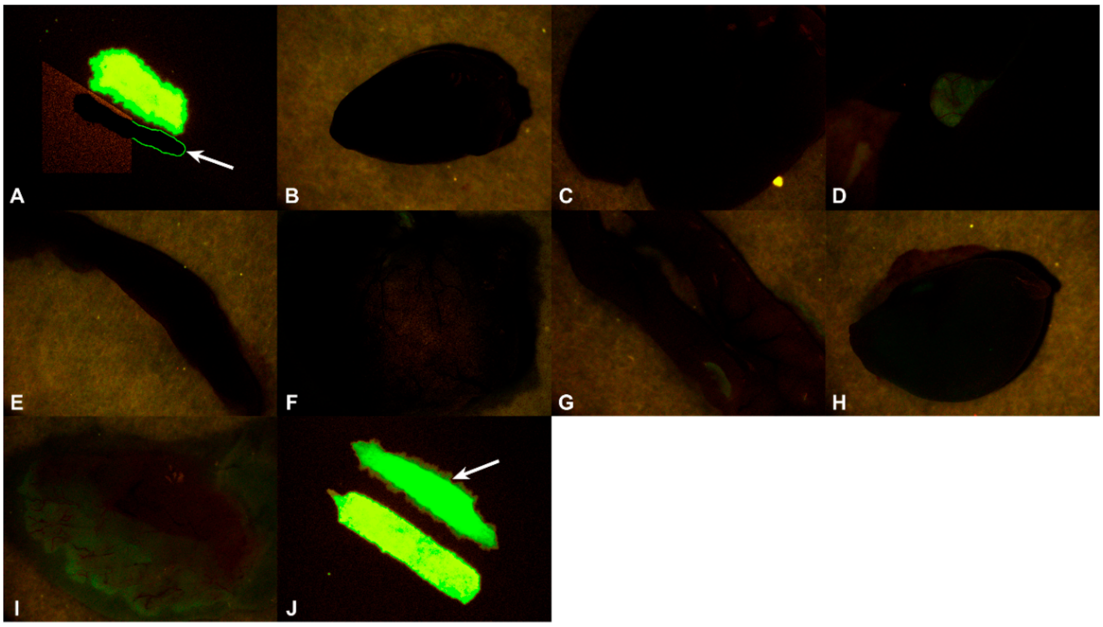

Figure S16. Fluorescence of CF-encapsulating DSPC:DSPE-PEG (96:4) LUVETs in (A) blood (arrow) and physiological buffer (intense green smear), (B) heart, (C) lungs, (D) liver and gallbladder, (E) spleen, (F) stomach, (G) intestines, (H) kidney, (I) epididymis, and (J) urine (arrow) and physiological buffer (intense green smear). Half of the blood smear in (A) was enhanced by augmenting the intensity by 300% to demonstrate the lack of CF fluorescence in blood. The other half of the smear was contoured. Organs were harvested approximately 60 min after LUVET infusion and imaged using an exposure time of 1.0 s and a gain of 5.0 ×.

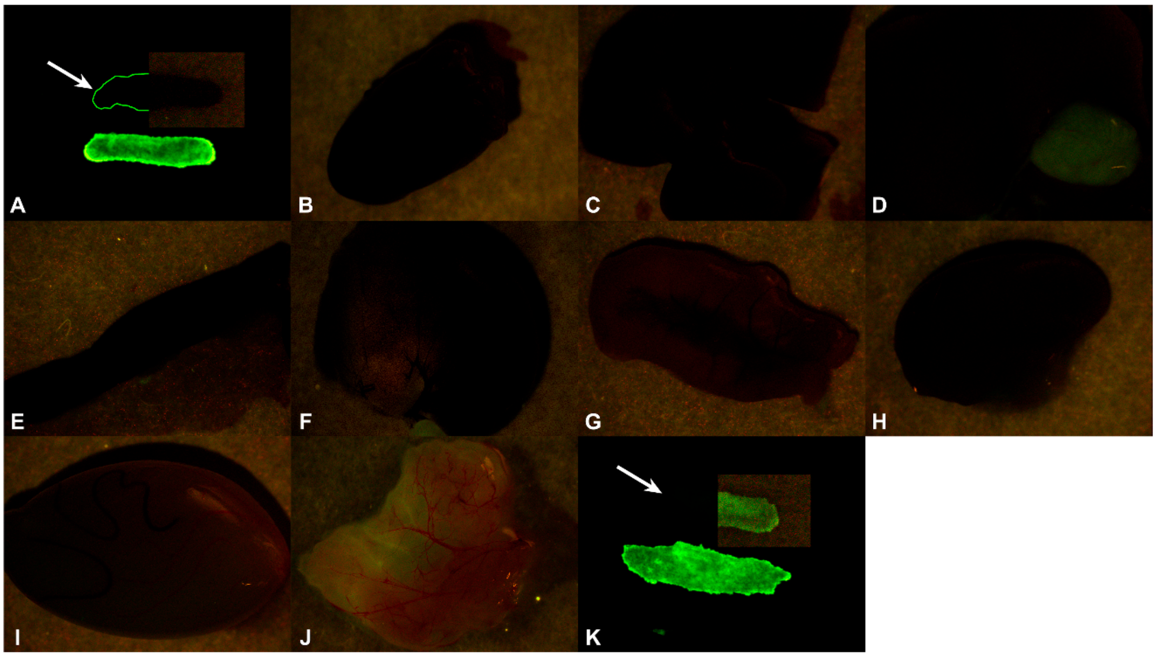

Figure S17. Fluorescence of NBD-labeled DPPC:DSPE-PEG (96:4) LUVETs in (A) blood (arrow) and physiological buffer (intense green smear), (B) heart, (C) lungs, (D) liver and gallbladder, (E) spleen, (F) stomach, (G) intestines, (H) kidney, (I) testicle, (J) epididymis, and (K) urine (arrow) and antibody solution (intense green smear). Half of the blood smear in (A) was enhanced by augmenting the intensity by 375% to demonstrate the lack of NBD fluorescence in blood. The other half of the smear was contoured. Half of the urine smear in (K) was enhanced by augmenting the intensity by 375% to demonstrate NBD fluorescence in urine. Organs were harvested approximately 135 min after antibody infusion and imaged using an exposure time of 1.5 s and a gain of 5.0 ×.

References

1. Heger, M.; Salles, II; Bezemer, R.; Cloos, M.A.; Mordon, S.R.; Begu, S.; Deckmyn, H.; Beek, J.F. Laser-induced primary and secondary hemostasis dynamics and mechanisms in relation to selective photothermolysis of port wine stains. *J Dermatol Sci* **2011**, *63*, 139-147, doi:10.1016/j.jdermsci.2011.04.015.
2. Varga, Z.; Wacha, A.; Bota, A. Osmotic shrinkage of sterically stabilized liposomes as revealed by time-resolved small-angle X-ray scatteringThis article will form part of a virtual special issue of the journal, presenting some highlights of the 15th International Small-Angle Scattering Conference (SAS2012). This special issue will be available in early 2014. *Journal of Applied Crystallography* **2014**, *47*, 35-40, doi:doi:10.1107/S1600576713030513.
3. van Raath, M.I.; Weijer, R.; Nguyen, G.H.; Choi, B.; de Kroon, A.I.; Heger, M. Tranexamic Acid-Encapsulating Thermosensitive Liposomes for Site-Specific Pharmaco-Laser Therapy of Port Wine Stains. *J Biomed Nanotechnol* **2016**, *12*, 1617-1640, doi:10.1166/jbn.2016.2277.
4. Gaber, M.H.; Wu, N.Z.; Hong, K.; Huang, S.K.; Dewhirst, M.W.; Papahadjopoulos, D. Thermosensitive liposomes: extravasation and release of contents in tumor microvascular networks. *Int J Radiat Oncol Biol Phys* **1996**, *36*, 1177-1187, doi:10.1016/s0360-3016(96)00389-6.
5. Han, S.-M.; Na, Y.-G.; Lee, H.-S.; Son, G.-H.; Jeon, S.-H.; Bang, K.-H.; Kim, S.-J.; Lee, H.-J.; Cho, C.-W. Improvement of cellular uptake of hydrophilic molecule, calcein, formulated by liposome. *Journal of Pharmaceutical Investigation* **2018**, *48*, 595-601, doi:10.1007/s40005-017-0358-0.
6. Mulik, R.; Kulkarni, V.; Murthy, R.S. Chitosan-based thermosensitive hydrogel containing liposomes for sustained delivery of cytarabine. *Drug Dev Ind Pharm* **2009**, *35*, 49-56, doi:10.1080/03639040802178144.
7. Dicko, A.; Kwak, S.; Frazier, A.A.; Mayer, L.D.; Liboiron, B.D. Biophysical characterization of a liposomal formulation of cytarabine and daunorubicin. *Int J Pharm* **2010**, *391*, 248-259, doi:10.1016/j.ijpharm.2010.02.014.

8. Lim, S.K.; Shin, D.H.; Choi, M.H.; Kim, J.S. Enhanced antitumor efficacy of gemcitabine-loaded temperature-sensitive liposome by hyperthermia in tumor-bearing mice. *Drug Dev Ind Pharm* **2014**, *40*, 470-476, doi:10.3109/03639045.2013.768631.
9. Tong, Q.; Li, H.; Li, W.; Chen, H.; Shu, X.; Lu, X.; Wang, G. In vitro and in vivo anti-tumor effects of gemcitabine loaded with a new drug delivery system. *J Nanosci Nanotechnol* **2011**, *11*, 3651-3658, doi:10.1166/jnn.2011.3804.
10. Liu, J.J.; Hong, R.L.; Cheng, W.F.; Hong, K.; Chang, F.H.; Tseng, Y.L. Simple and efficient liposomal encapsulation of topotecan by ammonium sulfate gradient: stability, pharmacokinetic and therapeutic evaluation. *Anticancer Drugs* **2002**, *13*, 709-717, doi:10.1097/00001813-200208000-00005.
11. Dadashzadeh, S.; Vali, A.M.; Rezaie, M. The effect of PEG coating on in vitro cytotoxicity and in vivo disposition of topotecan loaded liposomes in rats. *Int J Pharm* **2008**, *353*, 251-259, doi:10.1016/j.ijpharm.2007.11.030.
12. Lim, C.B.; Abuzar, S.M.; Karn, P.R.; Cho, W.; Park, H.J.; Cho, C.W.; Hwang, S.J. Preparation, Characterization, and In Vivo Pharmacokinetic Study of the Supercritical Fluid-Processed Liposomal Amphotericin B. *Pharmaceutics* **2019**, *11*, doi:10.3390/pharmaceutics11110589.
13. Moribe, K.; Tanaka, E.; Maruyama, K.; Iwatsuru, M. Enhanced encapsulation of amphotericin B into liposomes by complex formation with polyethylene glycol derivatives. *Pharm Res* **1998**, *15*, 1737-1742, doi:10.1023/a:1011912829747.
14. Javed, I.; Hussain, S.Z.; Ullah, I.; Khan, I.; Ateeq, M.; Shahnaz, G.; Rehman, H.U.; Razi, M.T.; Shah, M.R.; Hussain, I. Synthesis, characterization and evaluation of lecithin-based nanocarriers for the enhanced pharmacological and oral pharmacokinetic profile of amphotericin B. *J Mater Chem B* **2015**, *3*, 8359-8365, doi:10.1039/c5tb01258a.
15. Li, L.; ten Hagen, T.L.; Hossann, M.; Suss, R.; van Rhoon, G.C.; Eggermont, A.M.; Haemmerich, D.; Koning, G.A. Mild hyperthermia triggered doxorubicin release from optimized stealth thermosensitive liposomes improves intratumoral drug delivery and efficacy. *J Control Release* **2013**, *168*, 142-150, doi:10.1016/j.jconrel.2013.03.011.
16. Maruyama, K.; Unezaki, S.; Takahashi, N.; Iwatsuru, M. Enhanced delivery of doxorubicin to tumor by long-circulating thermosensitive liposomes and local hyperthermia. *Biochim Biophys Acta* **1993**, *1149*, 209-216, doi:10.1016/0005-2736(93)90203-c.
17. Tagami, T.; Ernsting, M.J.; Li, S.D. Efficient tumor regression by a single and low dose treatment with a novel and enhanced formulation of thermosensitive liposomal doxorubicin. *J Control Release* **2011**, *152*, 303-309, doi:10.1016/j.jconrel.2011.02.009.
18. Shen, S.; Huang, D.; Cao, J.; Chen, Y.; Zhang, X.; Guo, S.; Ma, W.; Qi, X.; Ge, Y.; Wu, L. Magnetic liposomes for light-sensitive drug delivery and combined photothermal-chemotherapy of tumors. *J Mater Chem B* **2019**, *7*, 1096-1106, doi:10.1039/c8tb02684j.
19. Han, B.; Yang, Y.; Chen, J.; Tang, H.; Sun, Y.; Zhang, Z.; Wang, Z.; Li, Y.; Li, Y.; Luan, X., et al. Preparation, Characterization, and Pharmacokinetic Study of a Novel Long-Acting Targeted Paclitaxel Liposome with Antitumor Activity. *Int J Nanomedicine* **2020**, *15*, 553-571, doi:10.2147/IJN.S228715.
20. Yang, T.; Cui, F.D.; Choi, M.K.; Cho, J.W.; Chung, S.J.; Shim, C.K.; Kim, D.D. Enhanced solubility and stability of PEGylated liposomal paclitaxel: in vitro and in vivo evaluation. *Int J Pharm* **2007**, *338*, 317-326, doi:10.1016/j.ijpharm.2007.02.011.
21. Mayer, L.D.; Bally, M.B.; Loughrey, H.; Masin, D.; Cullis, P.R. Liposomal vincristine preparations which exhibit decreased drug toxicity and increased activity against murine L1210 and P388 tumors. *Cancer Res* **1990**, *50*, 575-579.
22. Tartau, L.; Cazacu, A.; Melnig, V. Ketoprofen-liposomes formulation for clinical therapy. *J Mater Sci Mater Med* **2012**, *23*, 2499-2507, doi:10.1007/s10856-012-4712-5.
23. Maestrelli, F.; Gonzalez-Rodriguez, M.L.; Rabasco, A.M.; Mura, P. Effect of preparation technique on the properties of liposomes encapsulating ketoprofen-cyclodextrin complexes aimed for transdermal delivery. *Int J Pharm* **2006**, *312*, 53-60, doi:10.1016/j.ijpharm.2005.12.047.
